# TIGER: A tdTomato in vivo genome-editing reporter mouse for investigating precision-editor delivery approaches

**DOI:** 10.1073/pnas.2506257122

**Published:** 2025-08-29

**Authors:** Samuel W. Du, Grazyna Palczewska, Zhiqian Dong, Julie C. Lauterborn, Balasankara Reddy Kaipa, Alexander L. Yan, Rafał Hołubowicz, Siyoung Ha, Paul Z. Chen, Christine M. Gall, Gulab Zode, David R. Liu, Krzysztof Palczewski

**Affiliations:** ^a^Gavin Herbert Eye Institute—Robert M. Brunson Center for Translational Vision Research, Department of Ophthalmology and Visual Sciences, University of California Irvine, Irvine, CA 92697; ^b^Department of Physiology and Biophysics, University of California Irvine, Irvine, CA 92697; ^c^Department of Anatomy and Neurobiology, School of Medicine, University of California Irvine, Irvine, CA 92697; ^d^Merkin Institute of Transformative Technologies in Healthcare, Broad Institute of Massachusetts Institute of Technology and Harvard, Cambridge, MA 02142; ^e^Department of Chemistry and Chemical Biology, Harvard University, Cambridge, MA 02142; ^f^HHMI, Harvard University, Cambridge, MA 02142; ^g^David H. Koch Institute for Integrative Cancer Research, Massachusetts Institute of Technology, Cambridge, MA 02139; ^h^Department of Chemistry, University of California Irvine, Irvine, CA 92697; ^i^Department of Molecular Biology and Biochemistry, University of California Irvine, Irvine, CA 92697

**Keywords:** base editing, prime editing, reporter, CRISPR, genome editing

## Abstract

In vivo genome editing has the potential to address many inherited and environmental disorders. However, a major hurdle for the clinical translation of genome editing is safe, efficient delivery to disease-relevant tissues. A modality-agnostic reporter animal model that facilitates rapid, precise, and quantifiable assessment of functional delivery and editing could greatly enhance the evaluation and translation of delivery technologies. Here, we present the development of the tdTomato in vivo genome-editing reporter (TIGER) mouse, a reporter strain that harbors an integrated and constitutively expressed mutated tdTomato gene in the *Polr2a* locus. The mutations (Q115X, Q357X) abolish fluorescence, but successful adenine base editing (ABE) or prime editing (PE) restores tdTomato fluorescence. This mouse model facilitates the tissue- and cell type–specific assessment of genome editing agent delivery. We describe several editing strategies validated in vitro and demonstrate efficient ABE and PE in vivo using viral and nonviral delivery vectors targeting four cell types within the mouse eye: the retinal pigment epithelium, photoreceptors, Müller glia, and the trabecular meshwork. We show direct editing characterization in the ocular tissues via in vivo and ex vivo two-photon confocal microscopy and verify the spectral and fluorescence lifetime properties of tdTomato reporter in other mouse tissues. Additionally, we demonstrate successful adeno-associated virus (AAV)-mediated PE of extraocular tissues, including hepatocytes, skeletal muscle, and brain neurons by intravenous injection. Thus, the TIGER mouse facilitates the direct development, comparison, and optimization of delivery platforms for efficient and productive ABE or PE broadly applicable in vivo across multiple tissues tested in this study.

## Significance

Genome editing in vivo could provide alternative therapeutic options for diseases with a genetic component. A critical step is the evaluation of delivery strategies for viral and nonviral genome editors. Current genome editing reporter models have shortcomings that limit their usefulness in translating findings about genome editor delivery. We developed a mouse model called tdTomato in vivo genome-editing reporter (TIGER) to serve as a reporter with single-cell resolution to evaluate and compare genome editing delivery methods across the mouse body. We demonstrate base and prime editing in seven therapeutically relevant cell types and four tissues. The TIGER mouse can serve as a useful model for benchmarking current delivery strategies and validating new approaches to advance genetic medicine.

Genome editing has the potential to ameliorate disease phenotypes and prevent disability and death from both inherited and acquired diseases. In particular, high-precision programmable CRISPR/Cas systems, such as base and prime editors, have been studied extensively for their ability to precisely target and edit genomic DNA in vitro and in animal models in vivo (1–3). A major challenge to translating CRISPR/Cas technologies into the clinic, however, is the issue of genome-editor delivery (4). The large size and complexity of some CRISPR/Cas systems, such as base editors and prime editors, challenge current state-of-the-art delivery technologies, such as viral vectors and lipid nanoparticles (LNPs) (5). Thus, the successful implementation of precision genome editing across all cell types and tissues necessitates the refinement of current delivery methods and the invention of new once.

To assess delivery and editing efficiency in vivo, the most direct and quantitative measurement is sequencing of the targeted locus (6, 7). However, this can only be performed when tissue is biopsied or the animal is killed. Additionally, sequencing of bulk tissues or cells obscures potential cell type–specific or spatial information about transduction and efficiency. DNA editing can also be context-dependent, with local sequences and epigenetic modifications altering editing efficiencies across different cells or tissues (8, 9). Another hurdle is the simultaneous optimization of both the delivery and editing strategies for novel targets, as low editing rates could be due to limited delivery or inefficient editing, or both, confounding the interpretation of the sequencing results.

Several mouse models have been utilized as general CRISPR reporters in vivo. Multiple groups have repurposed the Ai9 mouse to investigate CRISPR editing, though this strategy relies on multiple blunt double-stranded DNA breaks (DSBs) and indel formation to permit tdTomato expression (10–12). The mT/mG Cre-reporter mouse has also been repurposed in a similar fashion with single guide RNAs (sgRNAs) that target the loxP sites (13). In contrast to Cre reporters, multiple mouse models have been custom-designed to report on CRISPR activity. Early models utilized reporters with limited sensitivity, such as lacZ in whole tissues, and editing was undetectable in vivo (14). The majority of the other models report on nuclease Cas9 events (e.g., indel formation or homology-directed repair), utilizing fluorescent markers as readouts, such as the Traffic Light Reporter (TLR) and the ΔeGFP mice (15–17). While repairable by prime editor (PE), these are not addressable by a base editor (BE) and lack single-base precision in their reporting. Background autofluorescence from mouse tissues commonly overlaps in their fluorescence in the GFP channels, constraining the sensitivity and limit of detection (18). In contrast, imaging of tdTomato with longer wavelength excitation light is less prone to beam deterioration due to Rayleigh scattering. Finally, the insertion of the reporters into the *Rosa26* locus could also preclude the crossing of these reporter strains with other useful reporters or mouse models, such as the inducible loxP-PE2 mouse (19).

Another CRISPR mouse model, the luciferase LumA mouse, was recently reported to be a base and prime editable reporter for in vivo editing and was utilized to investigate systemic lipid-nanoparticle (LNP) delivery (20). This mouse also utilizes a single-nucleotide polymorphism to prevent expression when unedited, and functional luciferase is restored upon successful base (or prime) editing. While useful for investigating systemic biodistribution and organ-specific delivery, luciferase reporting lacks cell-type resolution and could obscure differences in cellular delivery.

Accordingly, we saw a need to develop a universal genome-editing reporter mouse that is responsive to both base editing and prime editing, both of which show great promise for precisely altering genomes and restoring normal physiology. We envisioned a single, ubiquitously expressed reporter that could be edited by different Cas9 derivatives and could be quantified rapidly at single-cell resolution. This reporter mouse could rapidly advance the development of delivery methods by enabling the characterization of functional biodistribution and quantitative evaluation of delivery modalities, such as identifying the most efficient AAV capsid, comparing viral versus nonviral delivery, or screening different compositions of LNPs. Here, we describe the generation and application of a tdTomato in vivo genome-editing reporter (TIGER) mouse line, which constitutively expresses an inactivated mutant tdTomato. We demonstrate the utility of the TIGER mouse by precisely documenting restored fluorescence in multiple tissues, including the eye, liver, muscle, and brain, upon delivery of base or prime editors by viral and nonviral methods.

## Results

### Conceptualization of tdTomato Reporter and Editing Strategies.

**Fig. 1.**
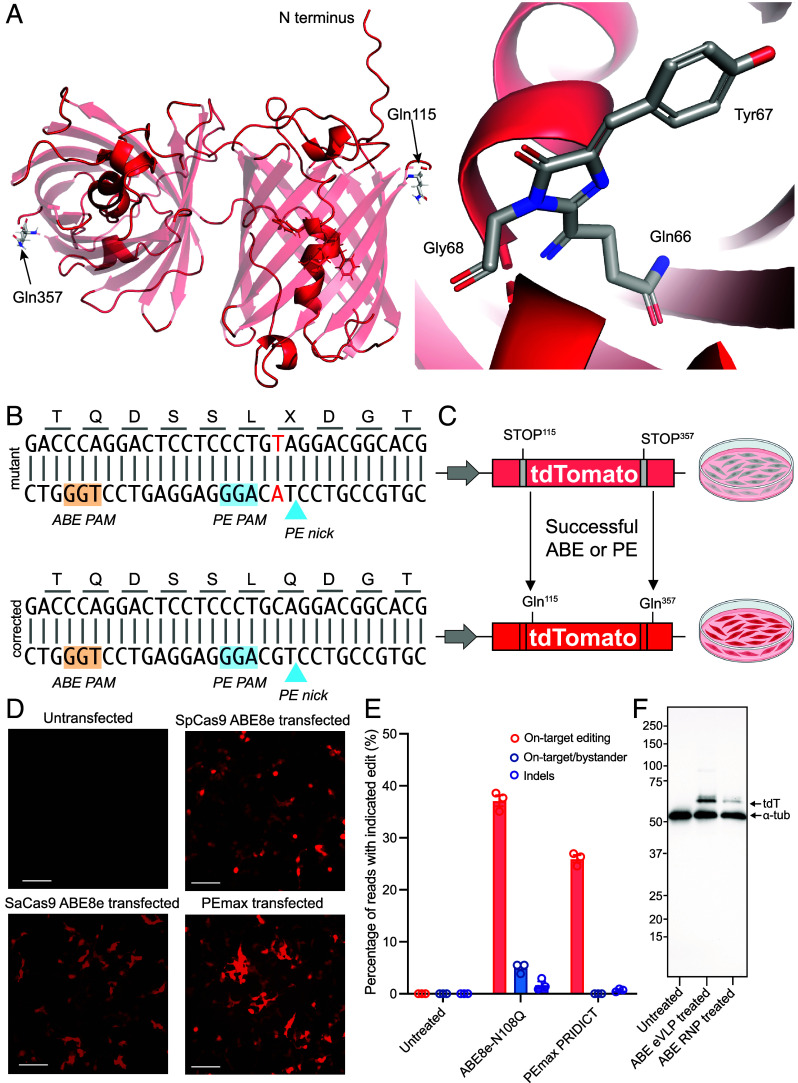
Validation of tdTomato editing strategies. (*A*) *Left*, AlphaFold2-predicted structure of tdTomato (23). The Gln115 and Gln357 residues, whose codons are replaced by nonsense TAG mutations, are represented in stick format on the ribbon diagram of the protein. *Right*, the *Inset* of ancestral DsRed chromophore structure determined by X-ray crystallography (24). (*B*) Genomic sequence of tdTomato. Upper sequence, mutant base in red; lower sequence, wild-type tdTomato. The PAM sequences for ABE (orange) and PE (blue) are highlighted. (*C*) Schematic diagram of TIGER construct stably expressed in HEK293T cells via lentiviral transduction. (*D*) Microscopy of TIGER HEK293T cells transfected with ABE or PE with cognate guide RNAs. (*E*) Amplicon sequencing of TIGER alleles by NGS, showing quantification of editing results. Mean ± SD. (*F*) Anti-α-tubulin and anti-RFP immunoblotting of HEK AAVS1 TIGER cell extracts obtained from cultures: 1) nontreated, 2) ABE RNP treated, 3) ABE eVLP treated. Predicted molecular weights of tdTomato: Nonedited, 13.1 kDa; N-terminal edited, 40.2 kDa; full-length, 54.2 kDa.

A critical challenge for the translation of genome-editing therapies is efficient in vivo delivery of genome-editing cargoes. In this regard, a robust reporter system would enable direct comparisons of delivery technologies targeted to different tissues. Accordingly, we sought to create a reporter mouse with characteristics that would facilitate the process of optimizing delivery modes, including 1) constitutive, pan-tissue, whole-body expression; 2) a nontoxic transgene; 3) a non-*Rosa26* locus to enable crossing of this mouse with other useful genome-editing transgenic mice; 4) easily detectable and quantifiable editing at the single-cell level; and 5) robust editability by multiple strategies, including base editing (BE) and prime editing (PE). We chose to investigate the use of tdTomato as a reporter. tdTomato is a tandem dimer of engineered red-fluorescent-protein (RFP) monomers, with a high quantum yield (0.69) (21) and is utilized in other mouse models, including ubiquitous expression in the mT/mG Cre reporter strain (22) (Fig. 1*A*). We reasoned that we could also select sites within tdTomato that were responsive to both adenine base editor (ABE) and PE by careful selection of residues that properly place the target base within the ABE editing window, as well as in close proximity to the DNA nick for PE. We selected codons which encode glutamine 115 and glutamine 357; mutation of the CAG codon to TAG where (C>T on the coding strand, G>A on the noncoding strand) leads to premature nonsense codons (Q115X and Q357X) that could be revertible by ABE and PE (Fig. 1*B*). Single base corrections by ABE or PE would then regenerate the CAG codon of Gln, leading to productive fluorescence. This design composes a useful reporter, which we term the TIGER (tdTomato in vivo genome editing reporter).

We created a TIGER HEK293T cell line to initially compare ABE and PE strategies in vitro by transducing HEK293T cells with a lentivirus encoding the mutant tdTomato (Fig. 1*C*). Before correction, the HEK293T cells were nonfluorescent; upon successful correction of the mutation, the HEK293T cells displayed red fluorescence. First, we confirmed that the mutant tdTomato construct was editable by *Streptococcus pyogenes* (Sp) Cas9 ABE. The most efficient editing, as assessed by flow cytometry, was effected by ABE8e-N108Q (25) with a sgRNA which places the mutated base at position 7 in the protospacer (A7) (*SI Appendix*, Fig. S1 *A* and *B*). We verified that other Cas9 variants, such as the *Staphylococcus aureus* Cas9 (SaCas9), could also successfully edit the mutant tdTomato (*SI Appendix*, Fig. S1*C*) (26). Then, we developed PE strategies to restore wild-type tdTomato by screening 2 pegRNA protospacers, each with 6 combinations of varying reverse-transcriptase templates (RTT) and prime-binding sites (PBS) (*SI Appendix*, Fig. S1*D*). We also utilized the PRIDICT algorithm to generate pegRNAs and tested these along with 3 nicking sgRNAs (*SI Appendix*, Fig. S1*E*) (27). Inclusion of these nicking sgRNAs led to a decrease in the proportion of tdTomato-positive cells by flow cytometry, no increase in the average editing efficiency, and an increase in indel rates, indicating these specific nicking sgRNAs were ineffective and suggesting that indels adversely affect restoration of tdTomato (*SI Appendix*, Fig. S1 *E* and *F*). Therefore, we opted to employ the PE2 strategy for PE correction of mutant tdTomato. Multiple editing strategies by SpCas9 and SaCas9 led to successful and precise correction of the tdTomato mutation, as assessed by microscopy and next-generation sequencing (NGS) deep sequencing (Fig. 1 *D* and *E*). Of note, editing of either monomer of the dimer is sufficient to result in tdTomato fluorescence, while editing of both monomers in the dimer results in increased fluorescence (*SI Appendix*, Fig. S2). Finally, Western blotting of lysates from TIGER HEK293T cells treated with SpCas9 ABE8e-N108Q RNP or SpCas9 ABE8e-N108Q eVLP confirmed the restoration of tdTomato protein in TIGER HEK293T cells (Fig. 1*F*).

### Design of the TIGER Mouse.

**Fig. 2.**
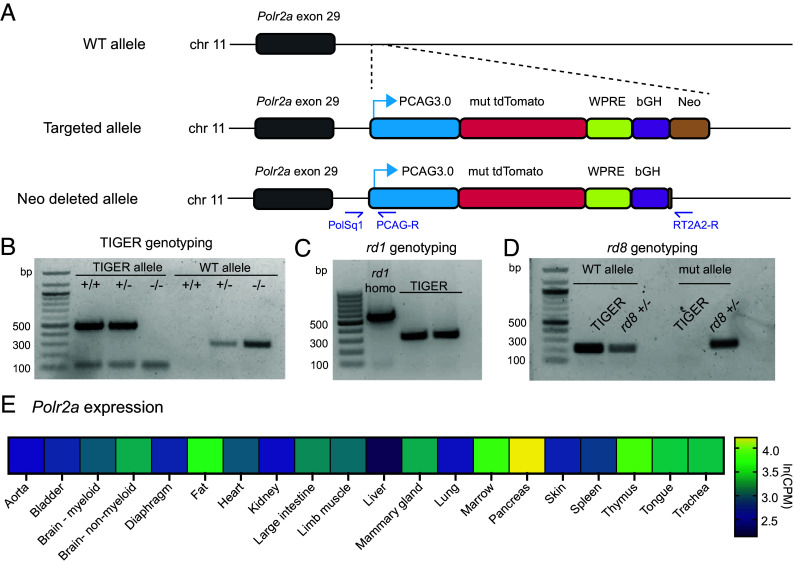
Design and construction of the TIGER mouse. (*A*) Strategy for knock-in of the mouse TIGER construct into the *Polr2a* locus. PCAG3.0, chicken beta-actin hybrid promoter; WPRE, woodchuck hepatitis virus posttranscriptional regulatory element; bGH, bovine growth hormone polyadenylation signal; Neo, neomycin resistance cassette. (*B*) TIGER genotyping gel. (*C*) Verification of absence of the *rd1* mutation. (*D*) Verification of absence of the *rd8* mutation. (*E*) Heatmap of *Polr2a* expression in mouse tissues by single-cell RNA sequencing [ln(CPM) of FACS data] from the Tabula Muris dataset (30).

After validating our construct and editing strategies in vitro, we used the same constructs to create our mice. As many useful reporter and transgenic mice are knocked into the *Rosa26* safe harbor locus, such as the Cre-inducible PE2 mouse (19), we instead knocked in our reporter cassette into the *Polr2a* locus on chromosome 11, which encodes a subunit of the DNA-dependent RNA polymerase and is widely expressed for DNA transcription. Other useful mouse lines, such as the RERT inducible Cre-ERT2 mouse [Jax #017585, (28)], and the GCaMP5G-IRES-tdTomato reporter mouse [Jax #024477, (29)] were previously knocked into the *Polr2a* locus, increasing our confidence in the selection of this alternative safe harbor site that would maintain compatibility with *Rosa26* mouse lines. In this construct, we knocked in the mutant tdTomato cassette, driven by the pCAG3.0 promoter, with the woodchuck hepatitis virus posttranscriptional response element (WPRE) and bovine growth hormone polyadenylation signal, along with a puromycin marker to facilitate clonal selection (Fig. 2*A*). After three backcrosses to C57BL/6J, we confirmed the genotype of our mice by PCR (Fig. 2*B*) and also confirmed that the mice were free of the retinal-degeneration-associated mutations *rd1* and *rd8,* which can occur in C57BL/6 lines (Fig. 2 *C* and *D*) (30, 31). We expected that this mutant tdTomato construct would be ubiquitously expressed throughout the mouse body, supported by reanalysis of SMART-seq2 single-cell RNA sequencing of multiple flow cytometry sorted tissues (Fig. 2*E*) (32).

### Spectral and Imaging Analysis of BE and PE in TIGER Eyes.

**Fig. 3.**
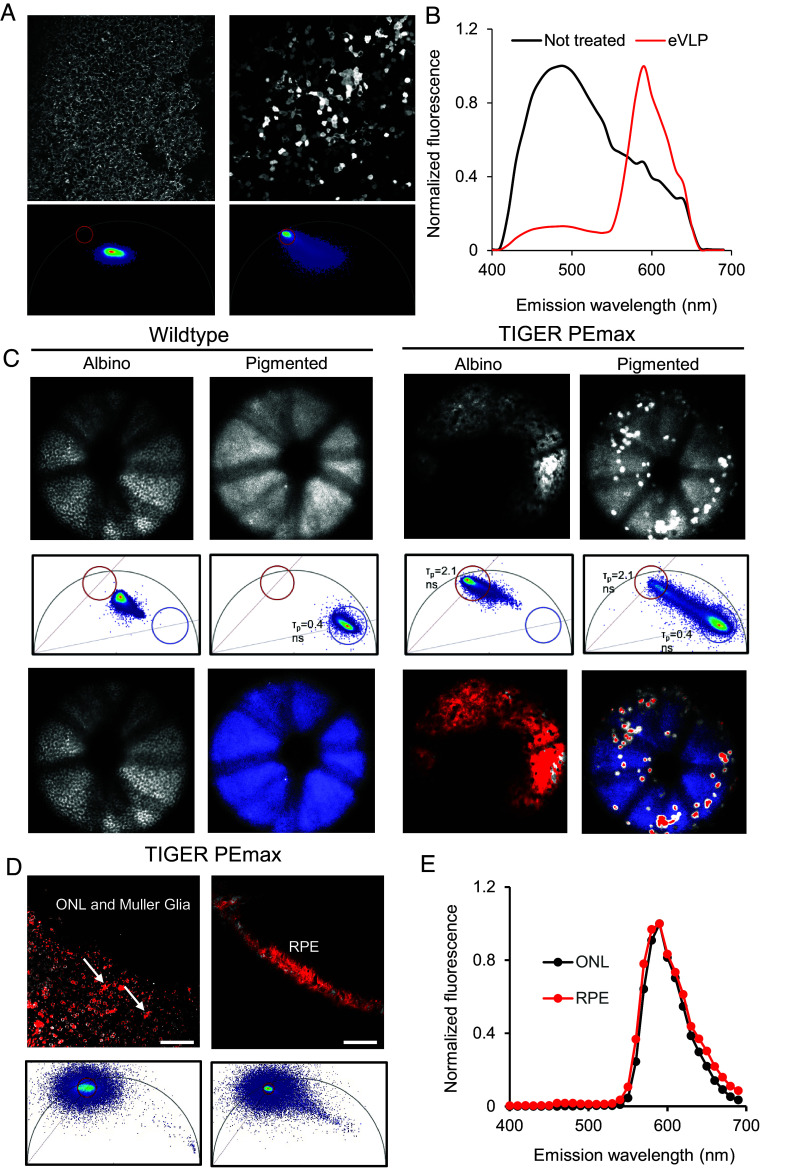
Two-photon characterization of tdTomato fluorescence properties in vivo. (*A*) Two-photon microscopy of HEK293T TIGER cells untreated or treated with ABE8e-N108Q eVLP. *Top*, fluorescence intensity signal; *Bottom*, phasor-plot FLIM analysis. The red circle in the phasor plots indicates the expected location of the tdTomato-FLIM signal. (*B*) Normalized emission spectrum of untreated (black) and eVLP-treated (red) cells. (*C*) Two-photon fundus imaging of anesthetized, live heterozygous TIGER mice either untreated (*Left*) or injected intravenously (*Right*) with dual-AAV2/8 PEmax. *Upper* panels, fluorescence intensity; *Middle* panels, phasor plot FLIM analysis; *Bottom* panels, FLIM in pseudocolor assigned by phasor plot analysis. (*D*) Phasor FLIM of different retinal cell types in the intact eyes of TIGER mice injected intravenously with dual-AAV2/8 PEmax. *Upper* panels, pseudocolor in FLIM (scale bar represents 50 µm); *Lower* panels, assignments in phasor plot analysis. (*E*) Normalized emission spectra of cells in the outer nuclear layer (ONL, black), and the RPE (red) in the eyes of PEmax-treated TIGER mice.

**Fig. 4.**
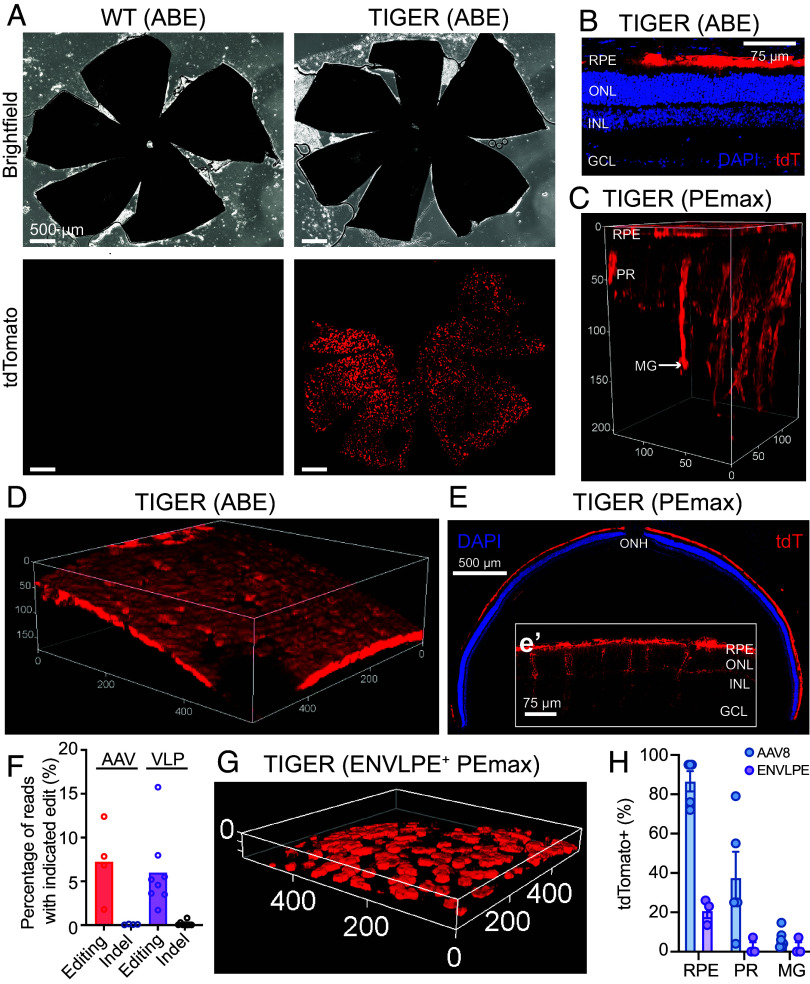
In vivo editing within the retinas of TIGER mice. (*A*) RPE flatmounts from the eyes of WT and TIGER mice treated subretinally with ABE-eVLP. (Scale bar represents 500 µm.) N = 14 for treated TIGER mice. (*B*) Microscopy of eye cryosection from an ABE-eVLP-treated TIGER mouse. (Scale bar represents 75 µm.) N = 14 for treated TIGER mice. (*C*) Two-photon 3D volumetric reconstruction of intact eye from a TIGER mouse treated with subretinal dual-AAV2/8 (scales provided in µm). RPE, retinal pigment epithelium; PR, photoreceptor; MG, Müller glia. N = 19 for treated TIGER mice. (*D*) Two-photon 3D volumetric reconstruction of the intact RPE of a TIGER mouse treated with subretinal ABE-eVLP (scales provided in µm). N = 14 for treated TIGER mice. (*E*) Microscopy of an eye cryosection from a TIGER mouse treated with subretinal dual-AAV2/8 PEmax. (Scale represents 500 µm.) The *Inset* is a magnified region of the cryosection. (Scale bar represents 75 µm.) ONH, optic nerve head. N = 19 for treated TIGER mice. (*F*) NGS amplicon sequencing analyzing precise correction and indel rates in four heterozygous TIGER eyes treated with dual-AAV2/8 PEmax (*Left*) and eight heterozygous TIGER eyes treated with ENVLPE+ PE VLPs (*Right*). (*G*) Two-photon 3D volumetric reconstruction of the intact RPE of a heterozygous TIGER mouse treated with subretinal ENVLPE^+^-PEmax-VLP (scales provided in µm). N = 8 for treated TIGER mice. (scales provided in µm). (*H*) Quantification via ImageJ analysis of maximum intensity projections of tdTomato+ cells per area in heterozygous TIGER mice treated with dual-AAV2/8 (blue, N = 5) or ENVLPE VLPs (purple, N = 3). RPE, retinal pigment epithelium; PR, photoreceptors; MG, Müller glia.

We first produced and purified VSV-G pseudotyped WT-SpCas9-ABE8e-N108Q eVLPs and confirmed that treated HEK293T TIGER cells restored fluorescence and exhibited tdTomato’s characteristic emission spectrum and fluorescence lifetime after treatment (Fig. 3 *A* and *B*). After successfully generating the TIGER mouse, we then transduced primary skin fibroblasts from heterozygous TIGER mice with the ABE8e-N108Q eVLPs and confirmed that the eVLPs corrected the mutation and restored fluorescence (*SI Appendix*, Fig. S3). Then, we proceeded to test in vivo base and prime editing of the retina and associated ocular tissues. We produced and purified WT-SpCas9 ABE8e-N108Q eVLPs (BE-eVLP) containing its cognate sgRNA that were validated above in vitro (25, 33). These loaded eVLPs were concentrated and injected subretinally into heterozygous TIGER mice and wild-type mice. After 1 wk, extensive tdTomato restoration was noted in heterozygous TIGER mice, but not in wild-type mice, when posterior eyecups, consisting of the retinal pigment epithelium (RPE), choroid, and sclera, were imaged as flatmounts (Fig. 4*A*). The restoration of tdTomato in the RPE was further corroborated by imaging of BE-eVLP-treated TIGER animals (Fig. 4*B*). This pattern of eVLP tropism corresponds to that previously reported for analogous eVLPs (34). To attempt productive editing of the photoreceptors, a major therapeutic target for inherited retinal degenerations (IRDs), we then packaged CAG-promoter-expressed SpCas9 PEmax and the U6-promoter-expressed tdT-PRIDICT-epegRNA into dual-AAV2/8 vectors in a previously described architecture (*SI Appendix*, Fig. S4), as the serotype is known to efficiently transduce RPE, photoreceptors, and Müller glia (1:1 ratio, 2.5 × 10^9^ viral genomes each) (35–37). Three weeks after subretinal injection of the dual-AAV2/8 vectors into heterozygous TIGER mice, we performed in vivo two-photon excitation and found that tdTomato is reproducibly and robustly detectable in imaging of live, anesthetized mice when probed with pulsed IR-light through the normal optical path of the anterior segment (cornea, lens, vitreous) (Fig. 3*C*). We then collected eyes for cryosectioning and found widespread transduction of the RPE, as well as expression in photoreceptors and Müller glia (Fig. 4*C*). These results were confirmed by two-photon imaging of intact TIGER eyes ex vivo and in vivo. Thus, 3D volumetric reconstruction, fluorescence lifetime (FLIM) analysis, and fluorophore spectral analysis showed robust PE-mediated tdTomato restoration in the RPE, photoreceptors, and Müller glia, all of which are postmitotic cells (Figs. 3 *D* and *E* and 4 *C* and *E*). Additional two-photon imaging and confocal microscopy of both intact and cryosectioned eyes showed that tdTomato restoration was robust and widespread in eyes treated with BE-eVLP (Fig. 4*D*) or with dual-AAV2/8 PEmax (Fig. 4*E*). When we sequenced bulk unsorted neural retinas treated with subretinal dual-AAV8 PEmax, we noted an average of 7.2% editing with minimal indel formation, as expected from a PE2 strategy (Fig. 4*F*). TIGER retinas are also editable through nonviral delivery of PEmax, as demonstrated by two-photon imaging and NGS of bulk RPE from TIGER mice treated by subretinal injection of ENVLPE+ PEmax-VLPs (Fig. 4 *F* and *G*) (38). Correlation of the editing rate was performed via fluorescence quantification of two-photon images (Fig. 4*H*). Sorting on tdTomato+ cells would likely increase our observed editing rates.

**Fig. 5.**
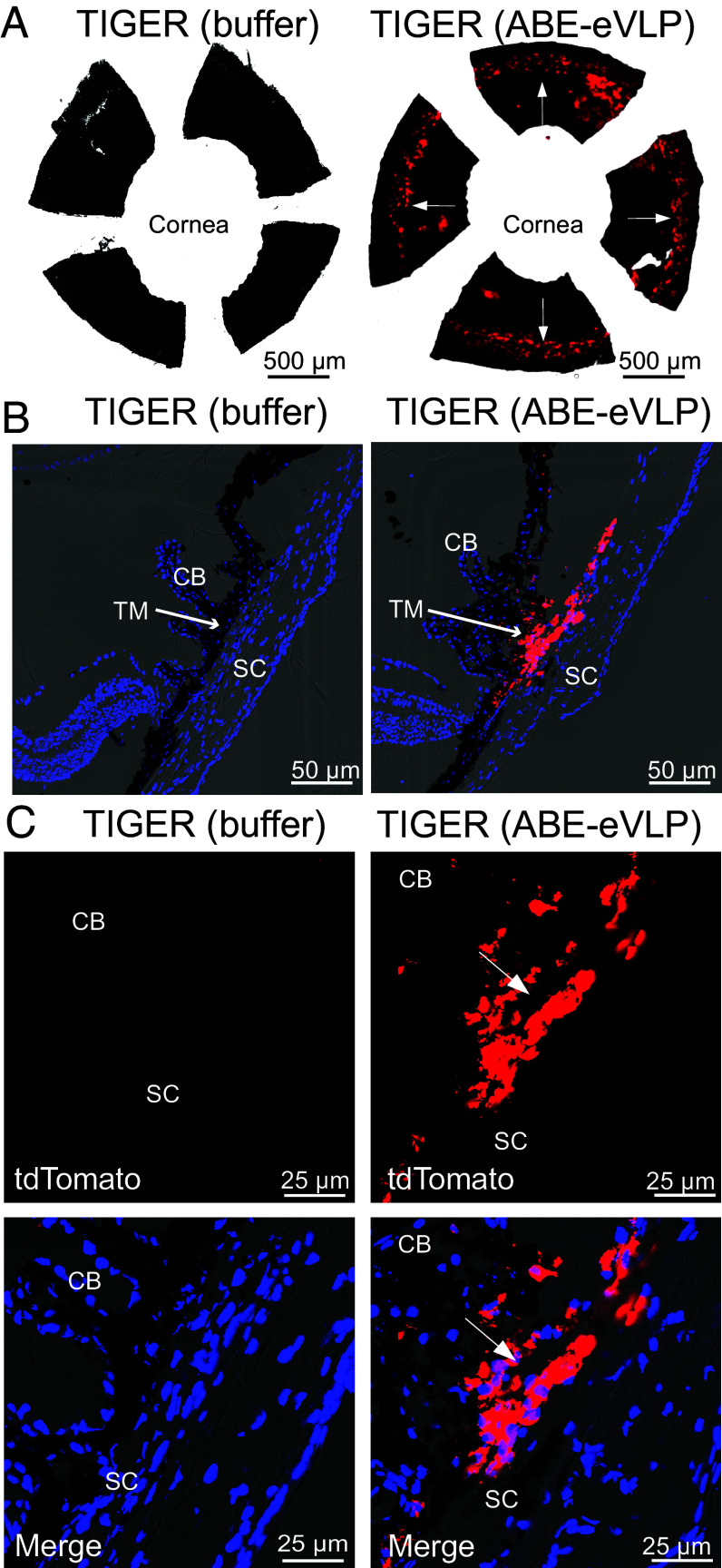
In vivo editing of the anterior segment from TIGER mice. (*A*) Anterior segment flatmount from a TIGER mouse injected intracamerally with ABE-eVLP- or buffer-treated control. White arrows point to the trabecular meshwork. (Scale bar represents 500 µm.) N = 3 for treated TIGER mice. (*B*) Low-magnification confocal imaging of cryosections of the anterior segment from a TIGER mouse treated with buffer, or injected intracamerally with ABE-eVLP. The white arrow points to the trabecular meshwork. CB, ciliary body; SC, Schlemm’s canal; TM, trabecular meshwork. (Scale bar represents 50 µm.) N = 7 for treated TIGER mice. DAPI, blue; tdTomato, red. (*C*) Higher magnification images of the sections shown in B. (Scale bar represents 25 µm.) DAPI, blue; tdTomato, red.

In the anterior segment, multiple cell types play a role in the pathology and progression of glaucoma. A major target for genome editing for glaucoma is the trabecular meshwork (TM), which regulates aqueous humor outflow and has been shown to be editable by nuclease Cas9 (39). Accordingly, we injected SpCas9 ABE-eVLP by intracameral infusion into the anterior chambers of the eyes of heterozygous TIGER mice and assessed tdTomato expression 1-wk posttreatment. Flatmounts of the anterior segment demonstrated robust tdTomato expression across the entire TM region (Fig. 5*A*). Microscopy of cryosectioned eyes from TIGER mice treated by intracameral injection of BE-eVLPs showed that the restoration of tdTomato was specific for the TM and not in the ciliary body (Fig. 5 *B* and *C*). These data demonstrate that the TM is amenable to efficient in vivo base editing, potentially opening the door for TM-targeted genome-editing therapies to treat glaucoma.

### Prime Editing in the Liver, Muscle, and CNS of TIGER Mice.

**Fig. 6.**
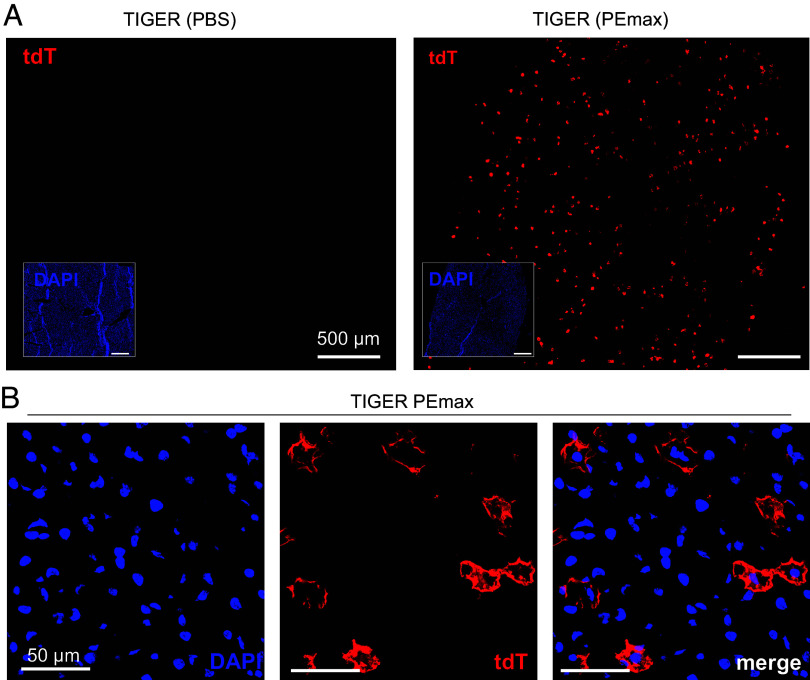
In vivo editing within the liver of TIGER mice. (*A*) Liver cryosections from TIGER mice injected intravenously with PBS (*Left*) or dual-AAV8 PEmax (*Right*), imaged for tdTomato. The *Insets* show the corresponding DAPI-counterstained sections. (Scale bar represents 500 µm.) N = 5 for treated TIGER mice. (*B*) High magnification liver cryosections from TIGER mice injected intravenously with dual-AAV8 PEmax. (Scale bar represents 50 µm.)

To investigate nonocular tissues in the TIGER mouse, we repurposed the dual-AAV2/8 SpCas9 PEmax vectors that were used to target the RPE, photoreceptors, and Müller glia. As AAV2/8 has been demonstrated to target liver tissues (33), we injected PBS or dual-AAV2/8 PEmax vectors (1:1 ratio, 2 × 10^11^ viral genomes each) intravenously via retro-orbital injection into heterozygous TIGER mice. Three weeks postinjection, we collected liver sections from mice injected either with PBS or with dual-AAV2/8 PEmax. As expected, we observed no tdTomato in PBS-injected TIGER mice, whereas the mice treated with dual-AAV2/8 PEmax displayed scattered tdTomato expression within the liver (Fig. 6 *A* and *B*).

**Fig. 7.**
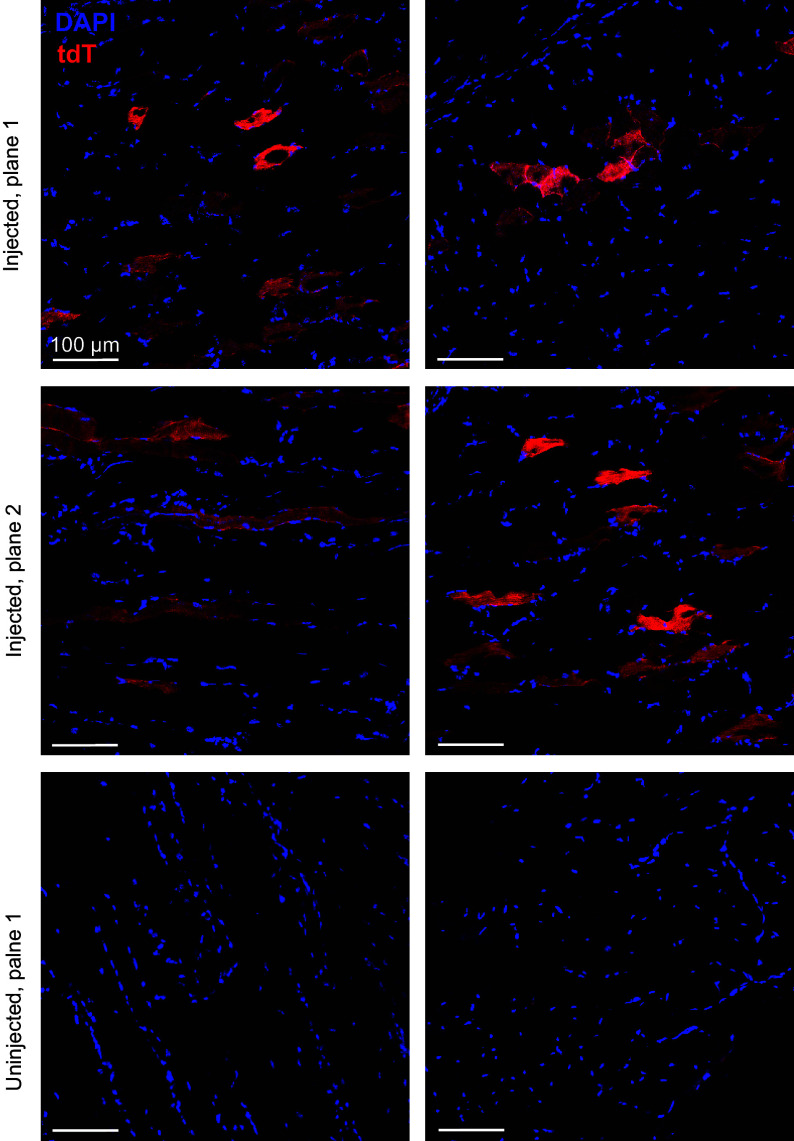
In vivo editing within the skeletal muscle of TIGER mice. Heterozygous TIGER mice were injected locally with 1 × 10^11^ VG of dual AAV8 PEmax into the right gastrocnemius muscle. Three weeks after injection, the treated-right and untreated-left gastrocnemius muscles were collected, sectioned, and imaged for tdTomato editing and fluorescent rescue. (Scale bar represents 100 µm.)

Additionally, we tested the editability of skeletal muscle myocytes. In addition to retinal and liver editing, AAV2/8 has also been previously utilized to deliver cargoes to skeletal muscle (40). Accordingly, we locally injected dual-AAV2/8 PEmax vectors (1:1 ratio, 1 × 10^10^ viral genomes each, 2 × 10^10^ viral genomes total) directly into the right gastrocnemius (calf) muscle of TIGER mice, while leaving the left gastrocnemius muscle as a control. Three weeks postinjection, we collected both gastrocnemius muscles and sectioned them to evaluate tdTomato expression. We noted tdTomato only in the treated, injected right gastrocnemius muscles, while the untreated comparator did not exhibit tdTomato fluorescence (Fig. 7). Depending on the plane at which the section was cut, we captured tdTomato-positive muscle fibers in either the short or long cross-section (Fig. 7).

A major target for genome editing is the neurons within the central nervous system (CNS). Extensive efforts have been made to develop delivery vehicles which bypass the blood–brain barrier (BBB) and deliver CRISPR/Cas9 systems to the CNS. One such AAV system is the PHP.eB capsid, which was evolved from AAV9 and AAV-PHP.B to efficiently cross the BBB (41). This capsid has previously been used to deliver BEs and PEs to the mouse brain (35, 42). Accordingly, we repackaged our PEmax AAV genomes into dual-AAV-PHP.eB vectors and injected them retro-orbitally (1:1 ratio, 1.875 × 10^11^ viral genomes each) into the systemic circulation. Three weeks postinjection, we collected brains and sectioned them to investigate the distribution of edited and labeled cells.

**Fig. 8.**
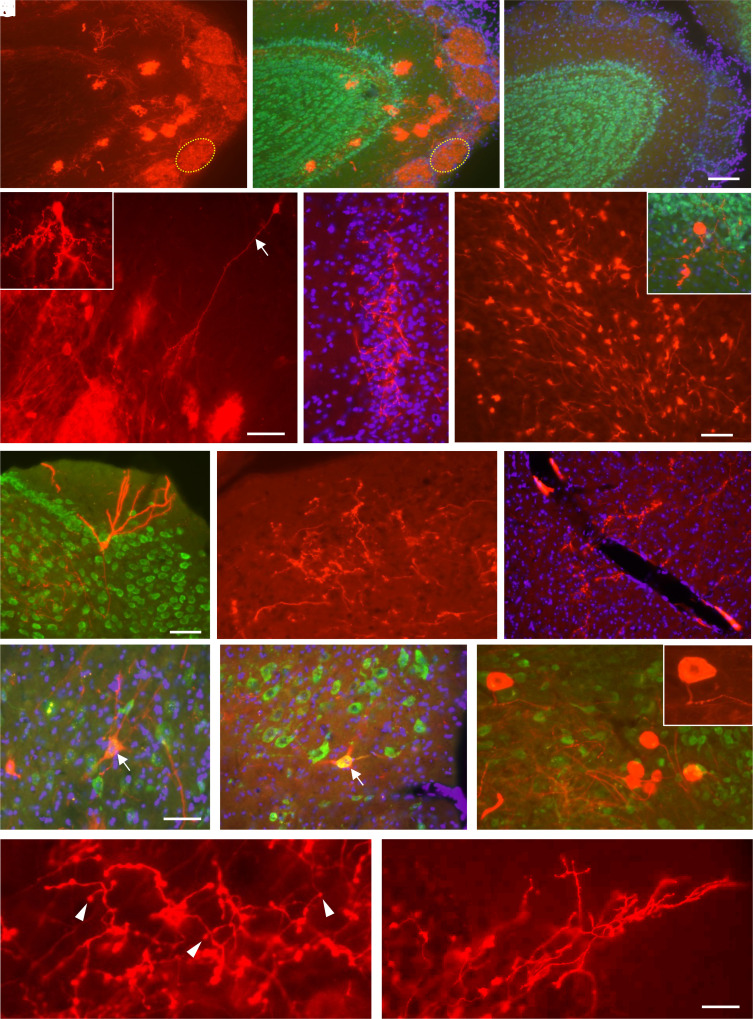
In vivo editing within the CNS of TIGER mice. (*A*–*D*) Images show tdTomato expression in the olfactory bulb of a TIGER mouse following injection of AAV (*A*, *B*, and *D*) or PBS (*C*); (*B*) shows neuronal layers labeled with NeuN (green) and DAPI (purple), relative to the tdTomato as shown in (*A*). The most prominent labeling following dual-AAV8 PEmax injection was in the glomeruli (dotted outline) and resident neurons including mitral cells and their processes (arrow); the *Inset* shows a mitral cell layer neuron. (*E*–*N*) Fields containing tdTomato expression in TIGER mice: (*E*) Anterior cingulate layer II; (*F*) cerebellar granule cell layer, with occasional cells in the Purkinje cell layer (*Inset*); (*G*) indusium griseum neuron; (*H*) axons in the cochlear nucleus; (*I*) axons in the deep inferior colliculus; (*J*–*L*) neuronal perikarya in the trigeminal motor (*J*), facial motor (*K*), and mesenchephalic trigeminal nuclei (*L*) (*Inset*, higher magnification); arrows indicate cells double-labeled for NeuN. (*M*) tdTomato-expressing mossy fibers in the interposed deep cerebellar nucleus (arrowheads); and (*N*) tdTomato-expressing climbing fibers extending into the cerebellar molecular layer. The pattern is typical for these types of axons which encase Purkinje cell dendrites. Scale bars: panel (*C*) = 100 µm for (*A*–*C*); panel (*D*) = 100 µm (50 µm for *Inset*); panels (*E* and *F*) = 40 µm (50 µm for *Inset*); panels (*G* and *I*) = 55 µm; panel (*H*) = 40 µm; panel (*J*) = 45 µm; panel (*K*) = 65 µm; panel (*L*) = 60 µm (45 µm for *Inset*); panel (*M*) = 15 µm; panel (*N*) = 45 µm. N = 5 for treated TIGER mice.

In TIGER mice treated with dual-AAV-PHP.eB, tdTomato-labeled neuronal perikaryal and processes were present in a number of regions. Robust labeling was evident in the olfactory bulb with expression in primary sensory afferent axons in the core of glomeruli and occasional resident neurons, including mitral and tufted cells (Fig. 8 *A*, *B*, and *D*); no expression was evident in this or any other brain region in control mice given PBS injections (Fig. 8*C*). Expression was rare in other forebrain regions in treated mice, although tdTomato-labeled axons were present in the superficial anterior cingulate (Fig. 8*E*) and deep somatosensory cortex, and an occasional labeled neuron was noted in the cortex, including in the indusium griseum (Fig. 8*G*). Greater tdTomato expression was present in the cerebellum, including labeling of numerous scattered cerebellar granule cells (Fig. 8*F*), a few cells in the Purkinje cell layer, occasional climbing fibers extending into the cerebellar molecular layer (Fig. 8*N*), and terminal mossy fiber afferents within the deep cerebellar nuclei (Fig. 8*M*). Brightly fluorescent axons were localized to the statoacoustic nerve and their termination in the cochlear nuclei (Fig. 8*H*) and scattered within the deep inferior colliculus (Fig. 8*I*) and mesencephalic reticular formation. Occasional tdTomato-expressing neuronal cell bodies were localized to cranial-nerve nuclei, including the trigeminal motor, facial motor, hypoglossal, spinal trigeminal, and mesencephalic trigeminal nuclei (Fig. 8 *J*, *K*, and *L*). Notably, within the brain, all labeling in treated mice appeared to be neuronal (perikaryal, axons, and dendrites); there was no evidence of tdTomato expression in nonneuronal cells nor was there any signal from neuronal or nonneuronal cells in the control, PBS-treated TIGER mice.

## Discussion

CRISPR/Cas9 and associated genome editing technologies have revolutionized basic research. They now facilitate the rapid targeted generation of mouse lines and cell lines and have been applied successfully to correct disease-causing mutations in animal models and in patients. As the capability and efficacy of genome editors have grown, there is an increased need for suitable delivery systems to correct genes in vivo. Certain cell types are directly accessible by local injection, such as the RPE and photoreceptors within the eye, while other cell types are readily accessible through systemic circulation, such as hepatocytes. For other organs and cell types that do not have well-developed delivery systems, we identified a need for a new reporter system that enables the evaluation and comparison of newly tailored delivery technologies. The TIGER reporter will enable the systematic assessment of ideal modalities for effective editing in vivo, as each cell and tissue type may require a customized solution for optimal efficacy.

We have successfully delivered SpCas9-based ABE and PE systems to seven cell types and four tissues within the mouse through viral and nonviral delivery methods to restore tdTomato fluorescence, as confirmed by imaging and spectral analysis, demonstrating the utility of this mouse model for assessing the efficacy and biodistribution of genome editing in vivo. While we only demonstrated SaCas9 editing in vitro, the TIGER mouse should also be amenable to editing by alternative Cas9 variants in vivo as well. The TIGER mouse will enable the direct comparison of different delivery modalities, including novel platforms, to therapeutically relevant cell types, with a detectable fluorescent signal within 1 wk of treatment. The signal is detectable in vivo in live mouse retina, eliminating the need to kill animals and prepare tissue samples. To further facilitate analysis, no staining or immunofluorescence is required. Therefore, we believe the TIGER mouse provides a reliable and sensitive reporter model with single-cell resolution to support the development of genome-editing therapy. During this study, we have evaluated three generations of TIGER mice for tdTomato editing and restoration with stable intensity and expression, though there is a chance that additional breeding could lead to epigenetic silencing of the promoter and locus. New delivery technologies can thus be compared and further translated into large animal models for IND-enabling studies.

Within the eye, we demonstrate base and prime editing of several major cell types of therapeutic interest. Many mutations which cause IRDs are found in the RPE, which we have previously targeted with VLPs and demonstrated cell-type specificity (33, 34). Indeed, when both ABE- and PE-VLPs were injected subretinally, we observed efficient conversion of the RPE, with some fields of view demonstrating over 90% conversion of this retinal cell layer. Another major cell type of interest in the retina is the retinal photoreceptors, which are most efficiently targeted by AAV delivery (43). Subretinal injection of dual-AAV2/8 PEmax particles resulted in efficient conversion of the RPE, photoreceptors, and Müller glia when imaged by two-photon confocal microscopy (44). Future development of photoreceptor-targeted viral and nonviral delivery methods evaluated in TIGER mice will advance the field of retinal genome editing. Notably, we also observed transduction of Müller glia with subretinal dual-AAV2/8. While few IRD-causing genes have been identified in Müller glia, some genes which cause IRDs, such as retinal G protein–coupled receptor (RGR), are expressed in Müller glia and could represent targets for gene therapy or genome editing (45, 46).

For genome editing clinical trials and therapeutic development, there is great interest in targeting hepatocytes for inherited diseases. One current target is transthyretin, which causes amyloidosis, with CRISPR-Cas9 delivered as mRNA LNPs (47). Another target is PCSK9 for the treatment of familial hypercholesterolemia, with adenine base editors delivered as mRNA LNPs (48). While our editing efficiency was modest after intravenous injection of dual-AAV2/8, we demonstrated proof-of-concept for hepatocyte delivery and expression across multiple liver lobes; other doses and delivery platforms can be tested and optimized for those who are interested in developing liver and hepatocyte-directed therapies.

With intravenous injection of the dual-AAV2/8 PEmax vectors, tdTomato was expressed in both sensory input and resident elements in the brain. On a global scale, tdTomato expression was relatively sparse and concentrated in specific areas. Specifically, tdTomato was robustly expressed in primary olfactory sensory axons and their field of termination in the core of the olfactory bulb glomeruli and within primary axons in the statoacoustic and trigeminal nerves. In each of these cases, the neuronal cell bodies are located outside the BBB, in peripheral ganglia, or, regarding the olfactory axons, in the nasal mucosa. In addition, tdTomato was robustly expressed by resident neuronal cell bodies including olfactory bulb mitral cells, cerebellar granule cells, and large neuronal perikaryal in several of the motor cranial nerve nuclei (i.e., the trigeminal motor, facial motor, and hypoglossal nuclei). In each of these instances, as is the case for neurons in the mesencephalic trigeminal nucleus, the labeled cells had processes that extend outside the BBB or reside in brain regions with notable BBB penetrance (olfactory bulb, cerebellum) (49, 50). Together, these results indicate that the AAVs were expressed by a variety of central neuronal cell types, giving rise to robust labeling through dendrites and, in some cases, lengthy axonal processes. Moreover, the regional distribution of labeling reflected brain areas and cells with greater access to the vector via the intravenous administration route. This suggests that other routes of administration that obviate the BBB would likely enable expression by other populations of neurons in areas such as hippocampus and striatum. Another alternative is to adjust dosing of the AAV—in one of our studies, we delivered 1 × 10^11^ VG for dual-AAV-mediated ABE for PCSK9 knockdown (33). In developing the dual AAV PE vectors, we delivered up to 1 × 10^12^ VG for systemic editing in liver and brain (35). Finally, in a recent paper where we used dual-AAV ABE to edit a prion gene, we delivered 3.75 × 10^12^ VG systemically (42). Taken together, these suggest that 2 × 10^11^ VG for our PE approaches are within the limits for effective editing, and there is room to adjust the titer or enhance AAV efficiency to boost our overall editing rates. Additionally, though we did not observe tdTomato editing and expression in glial cells, this is partially corroborated by other reports of PHP.eB tropism toward neuronal populations in the CNS (51, 52). While the editing of glial cells is an important problem, we believe that concerted additional focus on the specific application and optimization of base and prime editors to CNS glia will result in robust solutions and potential new therapies.

Looking to potential future applications of the TIGER mouse, we envision that it could easily be applied to the screening and validation of novel and engineered AAV capsids (53). The TIGER mouse can also be used to test the in vivo performance of the recently described evolved eVLPs (54). Experiments in the TIGER mouse could establish the feasibility of exon rewriting and cargo integration as described in cell culture in the PASTE (55), twin PE (56), and eePASSIGE platforms (57). Combined with the LumA mouse, the TIGER-LumA dual reporter mouse could offer both tissue- and cellular-resolution. For example, there is interest in the combinatorial and machine learning guided synthesis of lipids and lipidoids for LNP genome editing formulations (58, 59). After the creation of an LNP library, a first-pass analysis of organ biodistribution by LumA can be followed up with cell-type resolution biodistribution by TIGER. Thus, we anticipate that the TIGER mouse can rapidly validate advances in delivery technologies and provide a steppingstone between in vitro development of novel therapeutics and animal disease models.

## Materials and Methods

Assessment of tdTomato editing and fluorescence was carried out by next-generation sequencing, immunohistochemistry techniques, and two-photon excitation microscopy. Editing in the eye, brain, and liver of the TIGER mouse was performed with VLPs and AAVs. Additional details are available in *SI Appendix*.

### Mice and Ethics Statement.

C57BL/6J (“WT,” JAX 000664) and C57BL/6J albino (“WT albino,” JAX 000058) mice were purchased from the Jackson Laboratory (Bar Harbor, ME) and housed in the vivarium at the University of California, Irvine, where they were maintained on a normal mouse-chow diet and a 12 h/12 h light/dark cycle. All animals used in this study were between 1 and 3 mo of age. All animal procedures were approved by the Institutional Animal Care and Use Committee of the University of California, Irvine, and were conducted in accordance with the guidelines of the NIH for the care and use of laboratory animals, and with the Statement for the Use of Animals in Ophthalmic and Visual Research by the Association for Research in Vision and Ophthalmology.

## Supplementary Material

Appendix 01 (PDF)

## Data Availability

Amplicon sequencing data have been deposited in NCBI SRA under accession PRJNA1305119 (60). All other data are included in the manuscript and/or *SI Appendix*.
