# Supplementary material for: TIGER: A tdTomato in vivo genome-editing reporter mouse for investigating precision-editor delivery approaches
Source: Proc Natl Acad Sci U S A. 2025 Aug 29;122(35):e2506257122. doi: 10.1073/pnas.2506257122 (PMC12415246; doi:10.1073/pnas.2506257122)
Supplement: Supplementary file 1 — Appendix 01 (PDF) [file pnas.2506257122.sapp.pdf]

SUPPLEMENTAL INFORMATION

TIGER: A tdTomato *in-vivo* genome-editing reporter mouse for investigating precision-editor delivery approaches

Samuel W. Du[1,2,*], Grazyna Palczewska[1], Zhiqian Dong[1], Julie C. Lauterborn[3], Balasankara Reddy Kaipa[1], Alexander L. Yan[1], Rafał Hołubowicz[1], Siyoung Ha[1], Paul Z. Chen[4,5,6,7], Christine M. Gall[3], Gulab Zode[1], David R. Liu[4,5,6], Krzysztof Palczewski[1,2,8,9,*]

*To whom correspondence should be addressed:
Krzysztof Palczewski, Center for Translational Vision Research, Department of Ophthalmology , Gavin Herbert Eye Institute, UCI, Irvine, CA, 92697; phone (949) 824-6527; kpalczew@uci.edu
Samuel W. Du, Center for Translational Vision Research, Department of Ophthalmology and Visual Sciences, Gavin Herbert Eye Institute, UCI, Irvine, CA, 92697; phone (949) 824-5154; swdu@uci.edu

CONTENTS

Supplemental Methods
Supplemental Figure 1. Screening of tdTomato gene-correction strategies.
Supplemental Figure 2. Influence of the extent of TIGER editing on fluorescence of the reporter cells.
Supplemental Figure 3. eVLP-mediated transduction of primary TIGER mouse skin fibroblasts.
Supplemental Figure 4. Schematic diagrams of AAV constructs.
Supplemental Table 1. PCR primers.
Supplemental Sequences
Supplemental References

SUPPLEMENTAL METHODS

Generation of TIGER mice

TIGER mice were produced under a fee-for-service contract by Ingenious Targeting Laboratory (Holbrook, NY). **FLP** hybrid (129/SvEv x C57Bl/6 HF4) embryonic stem cells (ESCs) were electroporated with a linearized targeting vector containing 5' and 3' homology arms to *Polr2a*. The targeting vector contains PCAG3.0 driving mutant tdTomato, followed by the woodchuck post regulatory element (WPRE) and bovine growth hormone polyadenylation signal (bGHpA), with a FRT site flanked by a neomycin resistance cassette. After selection with G418, the clones were analyzed by PCR, and the neomycin cassette was removed during clone expansion. ESCs were injected into CD-1 blastocysts, and chimeras were mated with C57BL/6N at Ingenious. Upon receipt, the chimeras were then further backcrossed to C57BL/6J mice at least three times and screened for *rd1* and *rd8* mutations. Albino TIGER mice were additionally generated by backcrossing to C57BL/6J B6(Cg)-$Tyr^{c\text{-}2J}$/J albino mice (Jackson Laboratory #000058) for two-photon imaging experiments.

Genotyping

Ear punches from weaned mice were lysed in lysis buffer (Viagen Biotech # 402-E) with proteinase K (Viagen Biotech # 501-PK) overnight at 55 ºC. The samples were heat-inactivated at 85 ºC for 45 min, then debris was pelleted by centrifugation for 1 min at maximum speed in a tabletop microcentrifuge. The crude lysate was subsequently used in a standard endpoint PCR protocol, using GoTaq G2 Green Master Mix (Promega # M7822). PCR primers, cycling conditions, and amplicon sizes are described in Supplemental Table 1. Amplicons were analyzed by agarose gel electrophoresis, stained with SYBR Safe (Invitrogen # S33102) and compared to a molecular marker (Thermo Scientific # SM0241).

Cell culture and transfection

Naïve and tdTomato-stable HEK293T/17 cells were cultured in DMEM (Thermo Scientific # 10569010) with 10% heat-inactivated fetal bovine serum (Genesee Scientific # 25-514H) without antibiotics. The day before transfection, cells were seeded into 96- or 48-well plates to achieve 70-80% confluency the day of transfection. Plasmids were delivered with Lipofectamine 3000 (Thermo Scientific # L3000001), according to manufacturer instructions.

Plasmid preparation and molecular cloning

The fragment encoding wildtype tdTomato was amplified from pCA-mTmG (Addgene # 26123) and subcloned into pCMV-PE2 (Addgene # 132775) by USER cloning with Thermolabile USER II enzyme (NEB # M5508S). Mutations at Q115 and Q357 were introduced by KLD site-directed

mutagenesis (NEB # E0554S). Plasmids encoding sgRNAs and epegRNAs were generated as previously described by Golden Gate and Gibson assembly (1, 2).

Generation of the stable tdTomato cell line

The mutant tdTomato was subcloned into a lentiviral-genome vector with an IRES puromycin cassette for clone selection. Lentiviruses were produced by PEI max (Polysciences # 24765) triple transfection of the lentiviral genome, VSV-G envelope (Addgene # 8454), and psPAX2 (Addgene # 12260) into HEK293T/17 cells in complete media. 48 hours after transfection, lentiviral particles were precipitated with PEG-it solution (SBI Biosciences # LV810A-1). Naïve HEK293T/17 cells were then transduced with concentrated lentivirus, transduced cells were selected *via* puromycin selection, and clones were isolated by limiting dilution. Clones were screened by co-transfection of ABE and sgRNA plasmids.

Single versus double editing cell culture

TIGER HEK cells (clone 2E5, with TIGER construct integrated into AAVS1 site via PASSIGE) were seeded at 12,000 cells per well of a 96-well plate in DMEM, high glucose, pyruvate, GlutaMAX 1x (Thermo Fisher Scientific, 10569010) with 10% FBS without antibiotics. Twenty-four hours after seeding, the cells were transfected with 200 nM ABE8e N108Q NG (produced in-house) complexed with stabilized sgRNA targeting TIGER (Genscript) using 0.4 µL Lipofectamine 3000 per well (total volume: 100 µL) or transduced with ABE8e N108Q eVLP produced in house and concentrated by sucrose cushion ultracentrifugation 1,000x, at a final concentration of 20x relative to production culture. The medium was exchanged after 24 hr, and upon reaching confluence, the cells were passaged using 0.05% trypsin to 12-well plates and subsequently to 100-mm cell culture dishes into the same medium with additional 1% penicillin-streptomycin (Thermo Fisher Scientific, 15140122). Twelve days after transfection, the cells were sorted using BD FACSaria Fusion sorter according to the tdTomato fluorescence intensity and lysed in NGS lysis buffer (10 mM Tris-HCl pH 8.0, 0.05%(w/v) SDS, 20 µg/ml Proteinase K) at 1,000 cells/µL at 37 °C for 1 hr and subsequently at 85 °C for 45 min.

AAV preparation

The N-terminal PEmax AAV genome v3em-Nterm-PE2max was obtained from Addgene (# 198734). The epegRNA was cloned into C-terminal PEmax plasmid v3em-Cterm-PE2max-∆RNaseH-dualU6 (Addgene # 198735) by Gibson assembly (NEB # E2621S). Plasmids were propagated in NEB Stable *E. coli* (NEB # C3040H) and purified by endotoxin-free maxiprep (Thermo Scientific # K210016). AAV8 particles were packaged by the Penn Vector Core (RRID: SCR_022432) and AAV-PHP.eB particles were packaged by the UCI Center for Neural Circuit Mapping. The N- and C-terminal AAV8 titers were 3.81E12 GC mL^{-1} and 7.58E12 GC mL^{-1},

respectively. The N- and C-terminal AAV-PHP.eB titers were 1.46E13 GC mL^{-1} and 4.07E12 GC mL^{-1}, respectively. Vectors were stored at -80 ºC until use.

eVLP preparation

eVLPs were produced as previously described (3, 4). Briefly, eVLP plasmids were transfected into Gesicle HEK293 producer cells (Takara # 632617) with jetPRIME transfection reagent (Polyplus # 101000046); 48 hours after transfection, supernatants were collected, filtered and clarified, concentrated 1,000-fold by ultracentrifugation, and resuspended in PBS with 10% sucrose (w/v). Aliquots were stored at -80 ºC until use.

ENVLPE preparation

ENVLPE VLPs were produced as previously described (5). Briefly, ENVLPE production plasmids, following the protocol for ENVLPE+ particles, were transfected into Gesicle HEK293 producer cells (Takara # 632617) with jetPRIME transfection reagent (Polyplus # 101000046); 48 hours after transfection, supernatants were collected, filtered and clarified, concentrated 1,000-fold by ultracentrifugation, and resuspended in PBS with 10% sucrose (w/v). Aliquots were stored at -80 ºC until use.

Retro-orbital injections

Mice were anesthetized by isoflurane inhalation. Then, dual AAVs prepared aseptically were injected intravenously into the right retro-orbital sinus, using a 30G beveled needle.

Intra-muscular injections

Mice were anesthetized by isoflurane inhalation. Then, dual AAVs prepared aseptically in 30 μLof PBS were injected directly into the right gastrocnemius muscle, using a 30G beveled needle.

Subretinal and intravitreal injections

Subretinal and intravitreal injections were performed as previously described (6). Briefly, the eyes of the mice were bilaterally dilated, first with topical administration of 1% tropicamide ophthalmic solution (Akorn, 17478-102-12), followed by 10% phenylephrine ophthalmic solution (MWI Animal Health, 054243). Mice were then anesthetized by intraperitoneal administration of 20 mg mL^{-1} ketamine and 1.60 mg mL^{-1} xylazine in phosphate buffered saline (PBS) at a dose of 100 mg kg^{-1} of ketamine and 8 mg kg^{-1} of xylazine. To maintain corneal hydration, a drop of GenTeal Severe Lubricant Eye Gel was applied (0.3% hypromellose, Alcon). Subretinal and intravitreal injections were performed under an ophthalmic surgical microscope (Zeiss). For subretinal injections, using a 27G beveled needle, an incision was made in the cornea proximal to the limbus at the nasal side. A 34G needle with a blunt tip (World Precision Instruments, NF34BL-2), connected to an Nanofil injection holder (World Precision Instruments, NFINHLD) with SilFlex tubing (World Precision Instruments, SILFLEX-2), was inserted through the corneal incision into the anterior

chamber and advanced into the subretinal space without touching the lens. For intravitreal injections, using a 27G beveled needle, an incision was made immediately posterior to the ora serrata before a blunt 34G needle was inserted through the incision into the intravitreal space. Each mouse received a 1-μL injection in each eye at 70 nL s^{-1}, controlled by a UMP3 UltraMicroPump (World Precision Instruments, UMP3-4). After surgery, the mice were placed on a heating pad and anesthesia was reversed with intraperitoneal 2.5 mg kg^{-1} atipamezole in PBS (MWI Animal Health, #032800).

Intracameral injection procedure

Mice were anesthetized using an intraperitoneal injection of ketamine (100 mg kg^{-1}) and xylazine (10 mg kg^{-1}). Prior to injection, topical proparacaine HCl (0.5%) (Akorn Inc.) was applied to anesthetize the eyes. Additionally, 1% cyclopentolate (Mydriacyl®, Alcon Laboratories) was administered to induce pupil dilation.

A glass micropipette with a 35G beveled needle (World Precision Instruments, NF35BV) was used for injection, mounted onto a micro-dialysis infusion pump (World Precision Instruments, SP101I Syringe Pump) to deliver ABE-VLP at a controlled flow rate of 0.6 μL/min over 7–8 minutes. A total volume of 4 μL of either ABE-VLP or buffer (control) was administered *via* slow perfusion. Two weeks post-injection, mice were sacrificed, and their eyes were enucleated for further analysis of tdTomato.

Two-photon excitation

Two-photon excitation imaging was accomplished using our customized Leica TCS SP8 imaging system with Falcon architecture, enabling collection and analysis of fluorescence lifetime data. A pulse-selection system was incorporated for imaging of pigmented tissue, along with a periscope objective for live-mouse-eye imaging (7), a spectral detector, a 1.0 NA 20x water immersion objective, and a Vision S (Coherent) Ti:sapphire laser delivering 690-1050 nm 80-MHz pulsing light. To image tdTomato, an internal spectral detector was used with its detection bandwidths set to 590 - 680 nm for tdTomato. For *in vivo* imaging of anesthetized live mice, the pupil was dilated with 1% tropicamide ophthalmic solution (Akorn, 17478-102-12), followed by 10% phenylephrine ophthalmic solution (MWI Animal Health, 054243), and a 0-diopter contact lens was applied to protect the cornea from drying (8). *Ex vivo* intact mouse eyes were imaged after euthanasia and enucleation. Leica LASX 4.7.0.28176 and ImageJ (NIH) were used for the reconstruction of 3D stacks and quantification of transfected cells.(9, 10)

Retina tissue preparation and immunohistochemistry

Mice were killed and their eyes were enucleated and cleaned of excess tissue. The eyes were fixed for 20 min at RT in 4% paraformaldehyde (PFA) in PBS. Then, under a dissecting

microscope, the anterior segment was removed to form an eyecup. For flatmounts, radial cuts were made in the eyecup toward the optic nerve head, the retina and RPE were mechanically separated, and the flatmount was fixed for an additional 20 min at RT in 4% PFA in PBS. For cryosectioning, eyecups were fixed for an additional 20 min at RT in 4% PFA in PBS before sequential cryoprotection *via* increasing concentrations of sucrose in PBS before embedding in Tissue-Tek OCT (Sakura # 4583). Cryosections were cut at 12 µm. The flatmounts and cryosections were rehydrated in PBS with DAPI (1 µg ml^{-1}, Thermo Scientific # 62248), then mounted to a glass slide, and a coverslip affixed with Vectashield Antifade Mounting Medium containing DAPI stain (Vector Laboratories # H-1200-10). Images were acquired on a Keyence BZ-X810 fluorescence microscope or a Stellaris SP8 confocal microscope.

Anterior segment flatmount

To visualize tdTomato genome editing in the TM, anterior segment flat mounts were prepared. Enucleated eyes from VLP-ABE and control-injected TIGER mice were fixed in 4% paraformaldehyde and dissected to separate the anterior and posterior segments. The anterior segments were mounted on slides, cut into four quadrants to optimize flattening, and stained with a DAPI-containing mounting solution (VectaShield). Imaging was performed using a Keyence microscope (Itasca, IL, USA).

Liver and brain tissue preparation

Mice were deeply anesthetized and then perfused for 10 min with 4% PFA in PBS. Then, tissues were dissected from perfused animals. Liver samples were cut from three different liver lobes, placed into Tissue-Tek OCT, and cryosectioned, as described above.

Brains were postfixed for 2 h, cryoprotected in 20% sucrose/PB overnight, frozen and sectioned at 30 µm. To enhance visualization of tdTomato the slide-mounted tissue was processed for immunofluorescence using rat anti-mCherry (ThermoFisher Scientific # M11217) alone or in combination with guinea pig anti-NeuN (Synaptic Systems # 266014) for identification of neuronal nuclei (11). Secondary antisera included donkey anti-rat AlexaFluor 594 (ThermoFisher Scientific # A-21209), and goat anti-guinea pig AlexaFluor 488 (ThermoFisher Scientific # A-11073). Cover slips were affixed onto the sections on glass slides with Vectashield containing DAPI. Images were acquired using a Leica DM6000 epifluorescence microscope equipped with a Hamamatsu ORCA-ER digital camera and a Leica DM6000 with a sCMOS pco.edge digital camera.

Next-generation Illumina sequencing

Cultured cells were lysed as previously described (1) and bulk unsorted mouse tissues were processed with the Qiagen DNeasy Blood & Tissue kit (Qiagen #69504). The crude lysate or

purified DNA was used as the input for PCR1 using Phusion Plus polymerase (Thermo Fisher #F631S). PCR primers are described in **Supplemental Table 1**. One microliter of PCR1 was used as input for PCR2 to install Illumina barcodes. PCR2 was conducted for nine cycles of amplification using a Phusion HS II kit (Life Technologies). Following PCR2, samples were pooled, and gel-purified in a 1% agarose gel, using a QiaQuick Gel Extraction Kit (Qiagen # 28704). Library concentration was quantified using the Qubit High-Sensitivity Assay Kit (Thermo Fisher Scientific # Q32851). Samples were sequenced on an Illumina MiSeq instrument (paired-end read, read 1: 200–280 cycles, read 2: 0 cycles) using an Illumina MiSeq 300 v2 Kit (Illumina).

Long read sequencing

DNA containing TIGER locus was amplified using Phusion Plus Green PCR Master Mix (Thermo Fisher Scientific, F632L) and primers flanking the locus with a 35-cycle two-step protocol of denaturation at 98 °C for 10 sec and synthesis at 72 °C for 1 min, with initial denaturation at 98 °C for 30 sec and final synthesis at 72 °C for 5 min, in a final volume of 100 µL with 10 µL of the cell lysate as a template. PCR products were resolved on a 1.5% agarose gel in TAE, and ~1.7 kb band was purified from the gel using GeneJET Gel Extraction Kit (Thermo Fisher Scientific, K0691). Purified DNA was quantified by 1.5% agarose gel electrophoresis in TAE using GeneRuler 1 kb marker as a standard and submitted for long-read sequencing (premium PCR sequencing service, Plasmidsaurus). The reads were classified as non-edited, single- and double-edited (**Figure 1 c**) using an in-house Python script.

Western blotting

Bulk ABE-RNP and ABE-VLP treated and non-treated HEK TIGER cells were lysed in RIPA buffer (Cell Signaling Technology, 9806S) with protease inhibitors (cOmplete ULTRA EDTA-free, Roche, 5892953001), 200 µL per ~500,000 cells, for 1 hr in a cold room on a rotator, sonicated for 5 sec using a Qsonica 125W sonicator at 20% amplitude, and centrifuged for 20 min at 17,000*g* at 4 °C. The proteins were quantified with BCA assay. The samples were denatured using Laemmli sample buffer (Bio-Rad 1610747, 1x final concentration) with 60 mM dithtiothreitol by heating at 70 °C for 10 min, and applied on a discontinuous Tris-glycine-SDS 4% acrylamide (pH 6.8) – 10% acrylamide (pH 8.8) gel and resolved at 180 V for 45 min. The proteins were transferred onto 0.45 µm PVDF membrane (EMD Millipore, IPVH00010) using eBlot L1 system (Genscript), blocked for 1 hr in 2.5% non-fat milk in PBST, incubated in primary rabbit anti-RFP antibody (Rockland 600-401-379-RTU, 1:1,000) overnight and secondary anti-rabbit HRP-linked antibody (Cell Signaling Technology, 7074S, 1:2,000) for 1 hr, and developed using Pierce ECL plus chemiluminescent substrate (Thermo Fisher Scientific, FER32132). Blot was then washed and incubated with

1:2,000 anti-α-tubulin rabbit antibody (Cell Signaling Technology, 2144S) overnight and 1:5,000 anti-rabbit HRP for 1 hr, and developed accordingly.

Fibroblast culture and transduction

Newborn TIGER mice (P2) were sacrificed by decapitation, and their skins were isolated, washed in PBS with 100 U/ml penicillin and streptomycin and 40 μg/ml gentamicin, and fragmented into pieces which were adhered to a dry 150-mm tissue culture dish for 20 min in a cell culture incubator (37 °C, 5% CO_2). Then, the medium (DMEM/F12 with 15% FBS and 100 U/ml penicillin) was added onto the explants, and the fibroblasts were grown with media changes every 3 days. When approaching confluency, the fibroblasts were collected with TrypLE, filtered through a 70-micron cell strainer and seeded into a 48-well plate at 10,000 cells per well. After 24 hours, the ABE8e-N108Q eVLPs that were concentrated by ultracentrifugation 1,000x were diluted in the cell culture medium and applied onto the fibroblasts. After 46 hours, the medium was gradually exchanged to Fluorobrite DMEM with 1% FBS and the cells were imaged in a Keyence BZ-X810 microscope with red fluorescence filter (TxRed) and phase contrast. After imaging, the cells were washed with PBS, lysed with NGS lysis buffer and subjected to amplicon sequencing as described. Reads whose abundance was at least 0.10% were qualified for the analysis.

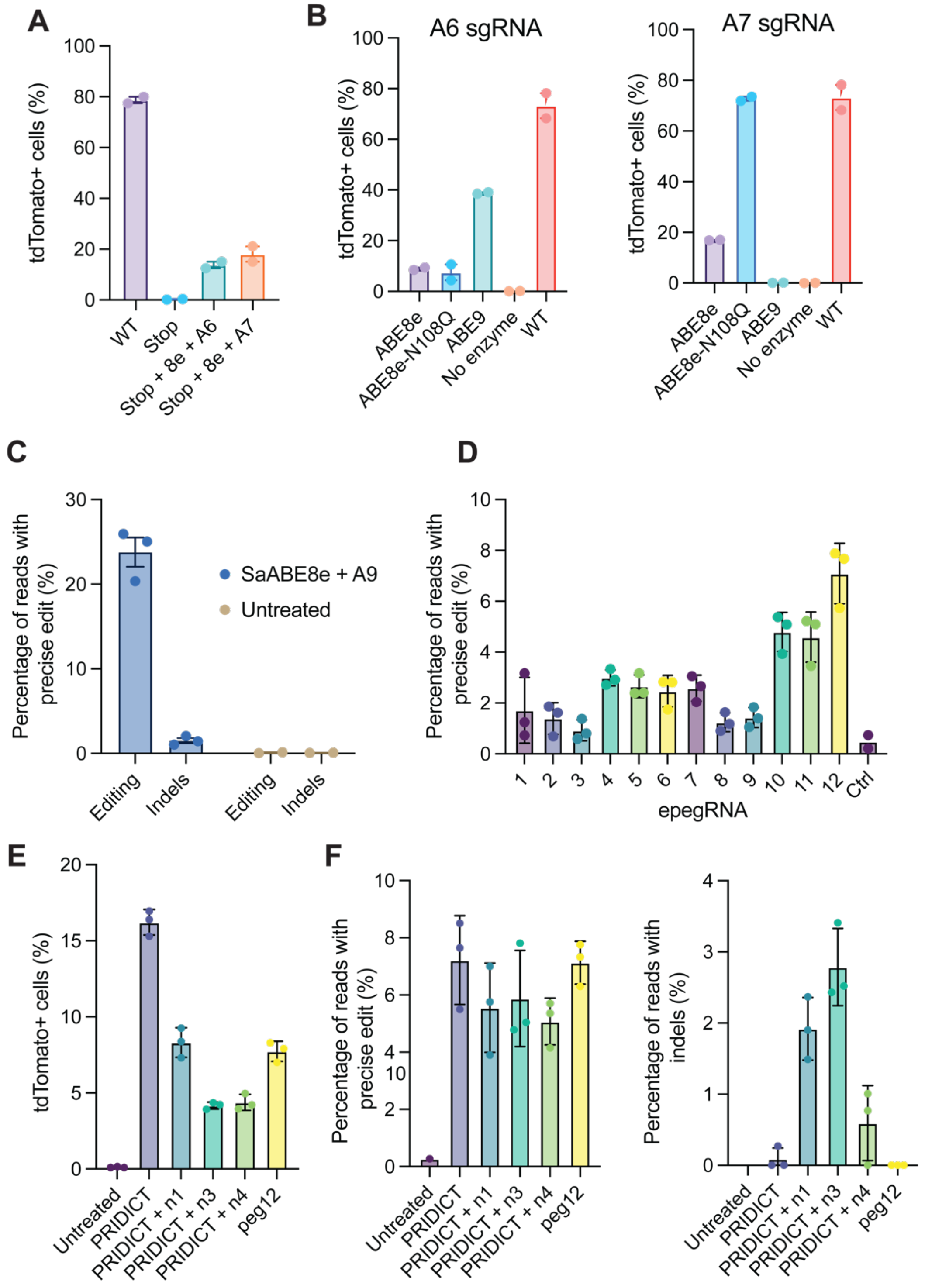

Supplemental Figure 1. Screening of tdTomato gene-correction strategies. (A) Flow-cytometry assessment of HEK293T TIGER cells: untransfected (Stop); transfected with wildtype tdTomato (WT), with ABE8e and A6 sgRNA (Stop + 8e + A6), or with ABE8e and A7 sgRNA (Stop + 8e + A7). (**B**) Flow-cytometry assessment of HEK293T TIGER cells treated with 3 ABE variants

and A6 sgRNA (left), or A7 sgRNA (right). (**C**) NGS sequencing of HEK239T TIGER cells transfected with SaCas9 ABE8e and A9 sgRNA. (**D**) NGS sequencing of HEK293T TIGER cells transfected with PEmax, along with each of 12 pegRNAs. (**E**) Flow cytometry of HEK293T TIGER cells transfected with PEmax and PRIDICT pegRNA, peg12, or each of 3 nicking sgRNAs (n1, n3, n4). (**F**) NGS sequencing of HEK293T TIGER cells transfected with PEmax and PRIDICT pegRNA, peg12, or each of 3 nicking sgRNAs (n1, n3, n4). Left, precise correction rate; right, indel rate.

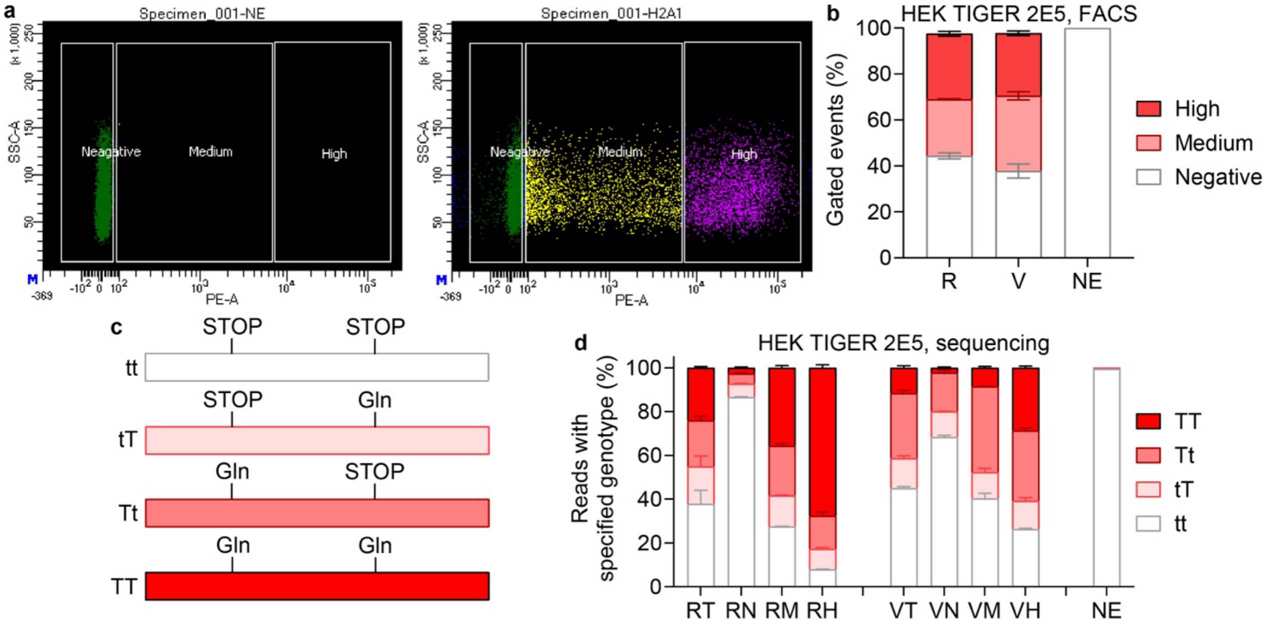

Supplemental Figure 2. Influence of the extent of TIGER editing on fluorescence of the reporter cells. (**a**) Representative distribution of fluorescence intensity of HEK TIGER cells. NE = no enzyme; H2A1 = ABE-RNP-treated; SSC = side scatter; PE = tdTomato fluorescence. (**b**) Fluorescence-activated cell sorting (FACS) classification of cells transfected with ABE-RNPs (R) or transduced with ABE-VLPs (V), with non-treated cells as a control. Two biological replicates for R and V, single sample for N, mean ± SD. (**c**) Schematic diagram of editing outcomes of the TIGER construct. (**d**) Editing outcome of TIGER construct in FACS-sorted cells, as reported by long-read sequencing. T = bulk cells; N = negative; M = medium; H = high tdTomato fluorescence. Two biological replicates for each sample, single sample for NE, mean ± SD.

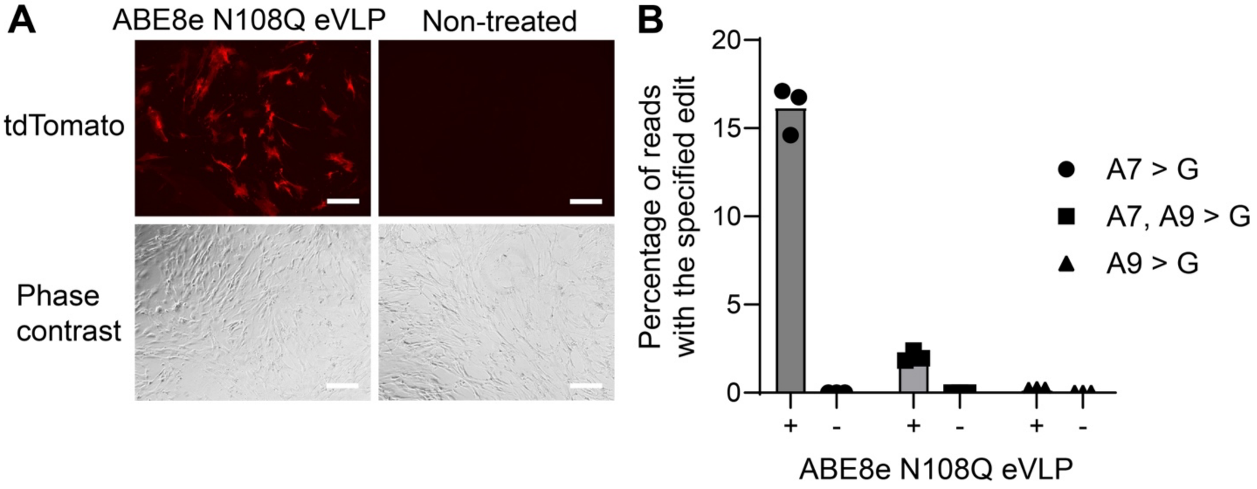

Supplemental Figure 3. eVLP-mediated transduction of primary TIGER mouse skin fibroblasts. (A) Representative red fluorescence (tdTomato) and phase contrast images of heterozygous TIGER mouse skin fibroblasts 46 hr after treatment with ABE8e N108Q VLP with sgRNA A7 concentrated by ultracentrifugation, at 16-fold concentration relative to VLP production media (scale bar represents 200 μm). (**B**) NGS sequencing of TIGER fibroblast genomic DNA after treatment with the ABE8e N108Q eVLP. Three biological replicates.

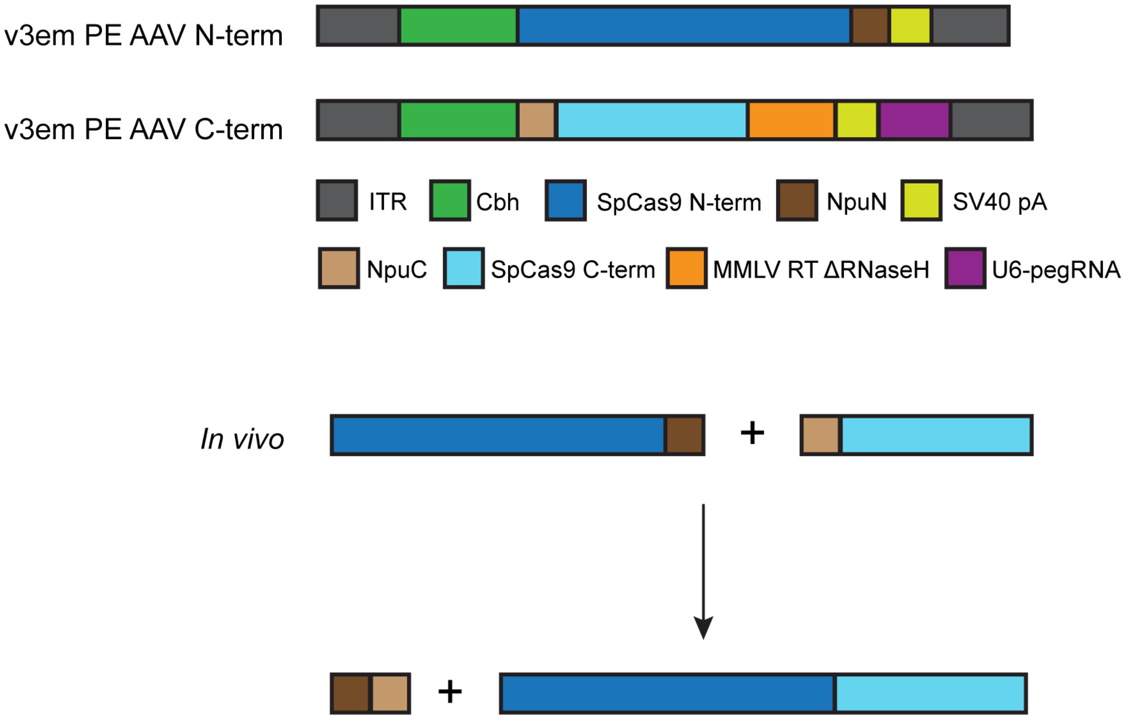

Supplemental Figure 4. Schematic diagrams of AAV constructs. After co-infection by both AAVs, the Npu self-splicing inteins recombine and reconstitute the full-length PEmax. ITR, inverted terminal repeats; Cbh, CMV early enhancer and modified chicken beta-actin promoter; SpCas9 N-term, *Streptococcus pyogenes* Cas9 N-terminus split at residue 1024; N-terminus NpuN, *Nostoc punctiforme* N-terminus self-splicing intein; SV40 pA, simian virus polyadenylation sequence; NpuC, *Nostoc punctiforme* C-terminus self-splicing intein; SpCas9 C-term, *Streptococcus pyogenes* Cas9 C-terminus split at residue 1024; MMLV RT ΔRNaseH, Moloney murine leukemia virus reverse transcriptase truncated at residue 497; U6-pegRNA, human nuclear U6 promoter driving epegRNA expression.

Supplemental Table 1. PCR primers and oligos.

Gene	*Primers (5' to 3')*	*Cycling parameters*		*PCR product size (bp)*
		Temp (°C)	*Time*	
TIGER mut	**Pol-Sq1-F:** tgaaccatctctccagcttcccagtcccc **POTP-PCAGREV**: TGACGTCAATGGAAAGTCCC	94<br>|94<br>35x |60<br>|72<br>72	2 min<br>30 sec<br>30 sec<br>60 sec<br>5 min	489
TIGER WT	**Pol-Sq1-F:** tgaaccatctctccagcttcccagtcccc **RT2A2REV:** CACTCTTAACAAAGAATTCATGTCTCTAGCTTAATT	94<br>|94<br>35x |60<br>|72<br>72	2 min<br>30 sec<br>30 sec<br>60 sec<br>5 min	315
Crb1 WT allele (separate reaction from *rd8* allele)	**mCrb1mF1:** GTGAAGACAGCTACAGTTCTGATC **mCrb1mR:** GCCCCATTTGCACACTGATGAC	94<br>|94<br>35x |65<br>|72<br>72	5 min<br>30 sec<br>30 sec<br>30 sec<br>7 min	220
Crb1 rd8 allele (separate reaction from WT allele)	**mCrb1mF2:** GCCCCTGTTTGCATGGAGGAAACTTGGAAGACAGCTACAGTTCTTCTG **mCrb1mR:** GCCCCATTTGCACACTGATGAC	94<br>|94<br>35x |65<br>|72<br>72	5 min<br>30 sec<br>30 sec<br>30 sec<br>7 min	244
rd1 WT allele (separate reaction from *mut* allele)	**Frd1common:** CATGTCCTACAGCCCCTCTC **Rrd1WT:** ACCATTTGCAAGGAAAGCAC	94<br>|94<br>35x |60<br>|72<br>72	2 min<br>30 sec<br>30 sec<br>60 sec<br>5 min	318
rd1 mut allele (separate reaction from WT allele)	**Frd1common:** CATGTCCTACAGCCCCTCTC **Rrd1MU:** AAGCTAGCTGCAGTAACGCCATTT	94<br>|94<br>35x |60<br>|72<br>72	2 min<br>30 sec<br>30 sec<br>60 sec<br>5 min	550
tdT-NGS-F	ACACTCTTTCCCTACACGACGCTCTTCCGATCTNNNNTGATATCTCACTTTGCTGCAGG			
tdT-NGS-R	TGGAGTTCAGACGTGTGCTCTTCCGATCTATGGCTAGACCATGAAGAAAGAAG			

pCMV-F (long read)	GCAAATGGGCGGTAGGC GTG			
IRES-R (long read)	CGGTGTGCGTTTGTCTATAT GTTATTTTCCAC			

Supplemental sequences
>TIGER_ABE_A7_sgRNA

cgtcctacagggaggagtccgttttagagctagaaatagcaagttaaaataaggctagtccgttatcaacttgaaaaagtggcaccga
gtcggtgc

>TIGER_SpCas9_epegRNA

GGATCAGCGTGCCGTCCTACGTTTTAGAGCTAGAAATAGCAAGTTAAAATAAGGCTAGTCCG
TTATCAACTTGAAAAAGTGGCACCGAGTCGGTGCAGGACTCCTCCCTGcAGGACGGCACG
CTGACGCGGTTCTATCTAGTTACGCGTTAAACCAACTAGAA

>tdTomato-STOP-DNA

atggtgagcaagggagaggaggtcatcaaagagttcatgcgcttcaaggtgcgcatggagggctccatgaacggccacgagttcg
agatcgagggcgagggcgagggccgcccctacgagggcacccagaccgccaagctgaaggtgaccaagggcggccccctgc
ccttcgcctgggacatcctgtcccccagttcatgtacggctccaaggcgtacgtgaagcaccccgccgacatccccgattacaaga
agctgtccttccccgagggcttcaagtgggagcgcgtgatgaacttcgaggacggcggtctggtgaccgtgacccaggactcctccct
gtaggacggcacgctgatctacaaggtgaagatgcgcggcaccaacttcccccccgacggccccgtaatgcagaagaagaccat
gggctgggaggcctccaccgagcgcctgtaccccgcgacggcgtgctgaagggcgagatccaccaggccctgaagctgaagg
acggcggccactacctggtggagttcaagaccatctacatggccaagaagcccgtgcaactgcccggctactactacgtggacacc
aagctggacatcacctcccacaacgaggactacaccatcgtggaacagtacgagcgctccgagggccgccaccacctgttcctgg
ggcatggcaccggcagcaccggcagcggcagctccggcaccgcctcctccgaggacaacaacatggccgtcatcaaagagttca
tgcgcttcaaggtgcgcatggagggctccatgaacggccacgagttcgagatcgagggcgagggcgagggccgcccctacgagg
gcacccagaccgccaagctgaaggtgaccaagggcggccccctgcccttcgcctgggacatcctgtcccccagttcatgtacggct
ccaaggcgtacgtgaagcaccccgccgacatccccgattacaagaagctgtccttccccgagggcttcaagtgggagcgcgtgatg
aacttcgaggacggcggtctggtgaccgtgacccaggactcctccctgtaggacggcacgctgatctacaaggtgaagatgcgcgg
caccaacttcccccccgacggccccgtaatgcagaagaagaccatgggctgggaggcctccaccgagcgcctgtaccccgcga
cggcgtgctgaagggcgagatccaccaggccctgaagctgaaggacggcggccgctacctggtggagttcaagaccatctacatg
gccaagaagcccgtgcaactgcccggctactactacgtggacaccaagctggacatcacctcccacaacgaggactacaccatcg
tggaacagtacgagcgctccgagggccgccaccacctgttcctgtacggcatggacgagctgtacaagtaa

>tdTomato-STOP-protein

MVSKGEEVIKEFMRFKVRMEGSMNGHEFEIEGEGEGRPYEGTQTAKLKVTKGGPLPFAWDILS
PQFMYGSKAYVKHPADIPDYKKLSFPEGFKWERVMNFEDGGLVTVTQDSSL*DGTLIYKVKMR
GTNFPPDGPVMQKKTMGWEASTERLYPRDGVLKGEIHQALKLKDGGHYLVEFKTIYMAKKPVQ

LPGYYYVDTKLDITSHNEDYTIVEQYERSEGRHHLFLGHGTGSTGSGSSGTASSEDNNMAVIKE
FMRFKVRMEGSMNGHEFEIEGEGEGRPYEGTQTAKLKVTKGGPLPFAWDILSPQFMYGSKAY
VKHPADIPDYKKLSFPEGFKWERVMNFEDGGLVTVTQDSSL*DGTLIYKVKMRGTNFPPDGPV
MQKKTMGWEASTERLYPRDGVLKGEIHQALKLKDGGRYLVEFKTIYMAKKPVQLPGYYYVDTK
LDITSHNEDYTIVEQYERSEGRHHLFLYGMDELYK*

>TIGER mouse genomic sequence

Yellow = 5’HA, green = PCAG3.0 promoter, red = mutated tdTomato, light blue = WPRE, green = bGH polyA, purple = 3’HA

ggtctcttgataattatgagccaccatgggggcattggaaaccaaatccatgttctctgacagaacaagtggggttggttggtttttagttta
agactagggtttctcagtatagccttggctaccctcagactccccagtgcagggattaaagcaccactaccaccagcccagaacaca
tgttggcctaattgctgaaccatctctccagcttcccagtccccattttgatatttttattctacacataggagaatacctgcctctatctctgga
atgctgggattatagacacatgccaccaaacctagccttataacttaatttttttagaaagatttttgccagtaggggtagcacacaaattta
ccagcactccagaggcagaggaaggcagagttgtacctggcctacatagtaacttccaggacagccagggctacatagtgaaatct
tgtctcaaaaaacaaagtttaatggcgcgccctcgacattgattattgactagttattaatagtaatcaattacggggtcattagttcatagc
ccatatatggagttccgcgttacataacttacggtaaatggcccgcctggctgaccgcccaacgacccccgcccattgacgtcaataat
gacgtatgttcccatagtaacgccaatagggactttccattgacgtcaatgggtggactatttacggtaaactgcccacttggcagtacat
caagtgtatcatatgccaagtacgccccctattgacgtcaatgacggtaaatggcccgcctggcattatgcccagtacatgaccttatgg
gactttcctacttggcagtacatctacgtattagtcatcgctattaccatgggtcgaggtgagccccacgttctgcttcactctccccatctcc
cccccctccccacccccaattttgtatttatttattttttaattattttgtgcagcgatgggggcgggggggggggggggcgcgcgccaggc
ggggcggggcggggcgaggggcggggcggggcgaggcggagaggtgcggcggcagccaatcagagcggcgcgctccgaa
agtttccttttatggcgaggcggcggcggcggcggccctataaaaagcgaagcgcgcggcgggcgggagtcgctgcgttgccttcg
ccccgtgccccgctccgcgccgcctcgcgccgcccgccccggctctgactgaccgcgttactcccacaggtgagcgggcgggacg
gcccttctcctccgggctgtaattagcgcttggtttaatgacggctcgtttcttttctgtggctgcgtgaaagccttaaagggctccgggagg
gccctttgtgcggggggggagcggctcgggggggtgcgtgcgtgtgtgtgtgcgtggggagcgccgcgtgcggcccgcgctgcccggc
ggctgtgagcgctgcgggcgcggcgcggggctttgtgcgctccgcgtgtgcgcgaggggagcgcggccgggggcggtgccccgc
ggtgcgggggggctgcgaggggaacaaaggctgcgtgcggggtgtgtgcgtgggggggtgagcagggggtgtgggcgcggcgg
tcgggctgtaacccccccctgcacccccctccccgagttgctgagcacggcccggcttcgggtgcggggctccgtgcggggcgtggc
gcggggctcgccgtgccgggcggggggtggcggcaggtgggggtgccgggcggggcggggccgcctcgggccggggagggct
cgggggaggggcgcggcggccccggagcgccggcggctgtcgaggcgcggcgagccgcagccattgccttttatggtaatcgtgc
gagagggcgcagggacttcctttgtcccaaatctggcggagccgaaatctgggaggcgccgccgcaccccctctagcgggcgcgg
gcgaagcggtgcggcgccggcaggaaggaaatgggcggggagggccttcgtgcgtcgccgcgccgccgtccccttctccatctcc
agcctcggggctgccgcaggggggacggctgccttcgggggggacggggcagggcggggttcggcttctggcgtgtgaccggcgg
ctctagagcctctgctaaccatgttcatgccttcttctttttcctacagctcctgggcaacgtgctggttattgtgctgtctcatcattttggcaaa
gaattcctagcttgggctgcaggtcgagggacctaaatcgatagctagcgctaccggactcagatctcttaattaacgcgtggatccat

gggttgctgtttctccaagaccatggtgagcaagggagaggaggtcatcaaagagttcatgcgcttcaaggtgcgcatggagggctc
catgaacggccacgagttcgagatcgagggcgagggcgagggccgcccctacgagggcacccagaccgccaagctgaaggtg
accaagggcggccccctgcccttcgcctgggacatcctgtcccccagttcatgtacggctccaaggcgtacgtgaagcaccccgcc
gacatccccgattacaagaagctgtccttccccgagggcttcaagtgggagcgcgtgatgaacttcgaggacggcggtctggtgacc
gtgacccaggactcctccctgtaggacggcacgctgatctacaaggtgaagatgcgcggcaccaacttcccccccgacggccccgt
aatgcagaagaagaccatgggctgggaggcctccaccgagcgcctgtaccccgcgacggcgtgctgaagggcgagatccacc
aggccctgaagctgaaggacggcggccactacctggtggagttcaagaccatctacatggccaagaagcccgtgcaactgcccgg
ctactactacgtggacaccaagctggacatcacctcccacaacgaggactacaccatcgtggaacagtacgagcgctccgagggc
cgccaccacctgttcctggggcatggcaccggcagcaccggcagcggcagctccggcaccgcctcctccgaggacaacaacatg
gccgtcatcaaagagttcatgcgcttcaaggtgcgcatggagggctccatgaacggccacgagttcgagatcgagggcgagggcg
agggccgcccctacgagggcacccagaccgccaagctgaaggtgaccaagggcggccccctgcccttcgcctgggacatcctgt
cccccagttcatgtacggctccaaggcgtacgtgaagcaccccgccgacatccccgattacaagaagctgtccttccccgagggct
tcaagtgggagcgcgtgatgaacttcgaggacggcggtctggtgaccgtgacccaggactcctccctgtaggacggcacgctgatct
acaaggtgaagatgcgcggcaccaacttcccccccgacggccccgtaatgcagaagaagaccatgggctgggaggcctccacc
gagcgcctgtaccccgcgacggcgtgctgaagggcgagatccaccaggccctgaagctgaaggacggcggccgctacctggtg
gagttcaagaccatctacatggccaagaagcccgtgcaactgcccggctactactacgtggacaccaagctggacatcacctccca
caacgaggactacaccatcgtggaacagtacgagcgctccgagggccgccaccacctgttcctgtacggcatggacgagctgtac
aagtaaaccggtcatcatcaccatcaccattgagtttaaacatttaaataatcaacctctggattacaaaatttgtgaaagattgactggt
attcttaactatgttgctccttttacgctatgtggatacgctgctttaatgcctttgtatcatgctattgcttcccgtatggctttcattttctcctccttg
tataaatcctggttgctgtctctttatgaggagttgtggcccgttgtcaggcaacgtggcgtggtgtgcactgtgtttgctgacgcaacccccc
actggttggggcattgccaccacctgtcagctcctttccgggactttcgctttccccctccctattgccacggcggaactcatcgccgcctg
ccttgcccgctgctggacaggggctcggctgttgggcactgacaattccgtggtgttgtcggggaagctgacgtcctttccatggctgctc
gcctgtgttgccacctggattctgcgcgggacgtccttctgctacgtcccttcggccctcaatccagcggaccttccttcccgcggcctgct
gccggctctgcggcctcttccgcgtcttcgccttcgccctcagacgagtcggatctccctttgggccgcctccccgcctgctccccgcctg
agagctcgctgatcagcctcgactgtgccttctagttgccagccatctgttgtttgcccctcccccgtgccttccttgaccctggaaggtgc
cactcccactgtcctttcctaataaaatgaggaaattgcatcgcattgtctgagtaggtgtcattctattctggggggtggggtggggcag
gacagcaagggggaggattgggaagacaatagcaggcatgctggggatgcggtgggctctatggcttctgaggcggaaagaacc
agctggggctcgatcctctagagcggccgccgtacgttcgtgggattgtgtccgtgtcgcgaagttcctatactttctagagaataggaa
cttcgttggtaccgtacgtaagctagagacatgaattctttgttaagagtgctcagtacacttggcagaggacccaagttcaatttccagc
atacaccaggcagtagtcacaactacccataagctccacctagcctccaacacagcctgcattcagtacacacacacatatataagtt
aaaggtcttttaaaaacaaaaacgggagttatggtgctggagagatagctcagggtaagagcagtgtattgctcttgaggggacctgg
gtccaggcacacacttggcacacatacatacatgaagacaagacactcagaagtaaatctataaaggggtcacctatttttattttctg
tgggtattcatgtctcagtgcattgtggtgccaggcctggctttcacatgaatgctggagacagcgcaagtcctgatgcttgaggggcag

acactaccaaaagaccaaaggaatcagctctctagctttccagtcacagctttgatttccgaagttacttcttgatgagaaatgtaactga
ctagaacaattcatctttgcc

SUPPLEMENTAL REFERENCES

1. Doman JL, Sousa AA, Randolph PB, Chen PJ, & Liu DR (2022) Designing and executing prime editing experiments in mammalian cells. *Nat Protoc* 17(11):2431-2468.
2. Huang TP, Newby GA, & Liu DR (2021) Precision genome editing using cytosine and adenine base editors in mammalian cells. *Nat Protoc* 16(2):1089-1128.
3. An M*, et al.* (2024) Engineered virus-like particles for transient delivery of prime editor ribonucleoprotein complexes in vivo. *Nat Biotechnol*.
4. Banskota S*, et al.* (2022) Engineered virus-like particles for efficient in vivo delivery of therapeutic proteins. *Cell* 185(2):250-265 e216.
5. Geilenkeuser J*, et al.* (2025) Engineered Nucleocytosolic Vehicles for Loading of Programmable Editors. *Cell* Accepted.
6. Du SW*, et al.* (2024) In vivo photoreceptor base editing ameliorates rhodopsin-E150K autosomal-recessive retinitis pigmentosa in mice. *Proc Natl Acad Sci U S A* 121(48):e2416827121.
7. Stremplewski P, Komar K, Palczewski K, Wojtkowski M, & Palczewska G (2015) Periscope for noninvasive two-photon imaging of murine retina in vivo. *Biomed Opt Express* 6(9):3352-3361.
8. Palczewska G, Kern TS, & Palczewski K (2019) Noninvasive Two-Photon Microscopy Imaging of Mouse Retina and Retinal Pigment Epithelium. *Methods Mol Biol* 1834:333-343.
9. Palczewska G*, et al.* (2014) Noninvasive two-photon microscopy imaging of mouse retina and retinal pigment epithelium through the pupil of the eye. *Nat Med* 20(7):785-789.
10. Palczewska G*, et al.* (2020) Noninvasive two-photon optical biopsy of retinal fluorophores. *Proc Natl Acad Sci U S A* 117(36):22532-22543.
11. Wang W*, et al.* (2018) Treating a novel plasticity defect rescues episodic memory in Fragile X model mice. *Molecular psychiatry* 23(8):1798-1806.
